# An LSC epigenetic signature is largely mutation independent and implicates the *HOXA* cluster in AML pathogenesis

**DOI:** 10.1038/ncomms9489

**Published:** 2015-10-07

**Authors:** Namyoung Jung, Bo Dai, Andrew J. Gentles, Ravindra Majeti, Andrew P. Feinberg

**Affiliations:** 1 Center for Epigenetics, Johns Hopkins University School of Medicine, Baltimore, Maryland 21205, USA; 2 Department of Medicine, Johns Hopkins University School of Medicine, Baltimore, Maryland 21205, USA; 3 Department of Medicine, Division of Hematology, Cancer Institute, and Institute for Stem Cell Biology and Regenerative Medicine, School of Medicine, Stanford University, Stanford, California 94305, USA; 4 Department of Radiology, and Center for Cancer Systems Biology, School of Medicine, Stanford University, Stanford, California 94305, USA

## Abstract

Acute myeloid leukaemia (AML) is characterized by subpopulations of leukaemia stem cells (LSCs) that are defined by their ability to engraft in immunodeficient mice. Here we show an LSC DNA methylation signature, derived from xenografts and integration with gene expression that is comprised of 71 genes and identifies a key role for the *HOXA* cluster. Most of the genes are epigenetically regulated independently of underlying mutations, although several are downstream targets of epigenetic modifier genes mutated in AML. The LSC epigenetic signature is associated with poor prognosis independent of known risk factors such as age and cytogenetics. Analysis of early haematopoietic progenitors from normal individuals reveals two distinct clusters of AML LSC resembling either lymphoid-primed multipotent progenitors or granulocyte/macrophage progenitors. These results provide evidence for DNA methylation variation between AML LSCs and their blast progeny, and identify epigenetically distinct subgroups of AML likely reflecting the cell of origin.

## Abstract (web summary)

Leukaemic stem cells are prevalent in acute myeloid leukemia. Here, Jung and colleagues derive a signature of 71 methylated genes that characterise these stem cells and find multiple *HOXA* genes within the signature.

## Graphical abstract

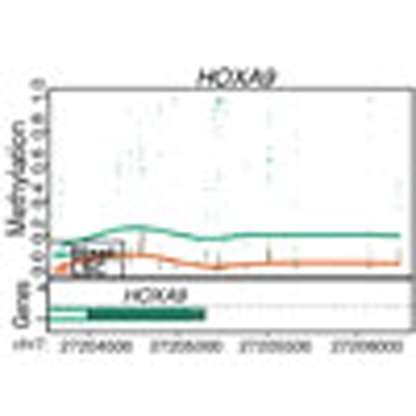

Acute myeloid leukaemia (AML) is an aggressive malignancy of bone marrow precursors defective in their maturation and function^1^. A large body of evidence indicates that like normal haematopoiesis, AML is organized as a cellular hierarchy initiated and maintained by a subpopulation of leukaemia stem cells (LSCs)^2^. These LSCs are functionally defined by their ability to transplant disease into immunodeficient mice, and are enriched in the immuno-phenotypically defined CD34+CD38− fraction of leukaemic cells^3^,^4^,^5^. AML LSCs in turn give rise to clonally related, downstream leukaemic blasts that lack engraftment potential. The clinical significance of this leukaemia stem cell model for AML is highlighted by the finding that LSC gene expression signatures are prognostic for poor outcome in multiple cohorts of AML patients^5^,^6^. As LSCs and their non-engrafting blast progeny are clonally related, a major implication of this leukaemia stem cell model is that their functional properties likely involve epigenetic differences. However, the epigenomic differences that would cause the functional differences between LSCs and their non-stem blast progeny have not been demonstrated experimentally. This would be a key addition to the previous literature since DNA methylation is stably copied during cell division in contrast to more labile patterns of gene expression^7^.

A number of both mouse and human studies have investigated the cell of origin in AML. Mouse studies have typically utilized retroviral oncogene transduction or knock-in models to explore this question and have generally led to the conclusion that committed progenitors, in particular common myeloid progenitors (CMP) and/or granulocyte/macrophage progenitors (GMP), serve as the cell of origin for most AML models. In one study of MN1-induced AML, retroviral transduction of single CMP, but not GMP or haematopoietic stem cells (HSC), resulted in the development of AML, indicating tight restriction of transformation by this oncogene^8^. In a second study using a mouse model of MLL-AF9 AML, the cell of origin influenced biological properties such as gene expression, epigenetics and drug responses^9^. Both of these studies highlight the significance of this question for leukaemogenesis and potential therapies. In contrast to mouse models, inferring the cell of origin in human leukaemia is only possible based on features of the disease. Studies investigating the cell of origin of human AML using surface immunophenotype and gene expression originally suggested AML LSCs arise from HSC^10^, but more recent analysis suggests they arise from committed progenitors, including lymphoid-primed multipotent progenitors (L-MPP) and GMP^3^. Notably, we and others have recently reported that leukaemogenic mutations arise in pre-leukaemic HSC that undergo further clonal evolution to give rise to AML LSC^11^,^12^,^13^, likely in downstream progenitors as has been demonstrated in chronic myeloid leukaemia (CML)^14^. Here we address this question of the cell of origin directly by determining the epigenetic signature of engrafting LSC in AML.

Dysregulation of the epigenome is a common feature in AML, as indicated by the recent discoveries that a number of epigenome-modifying genes are mutated in AML including some involved in the regulation of DNA methylation such as *IDH1/2*, *DNMT3A* and *TET2*, and modulation of chromatin modifications such as *ASXL1*, *EZH2* and others^15^,^16^. Beyond somatic mutations in these epigenome-modifying factors, initial characterization of DNA methylation in bulk AML cells revealed great heterogeneity among patient cases that could be clustered according to their methylation patterns^17^. In particular, AML with *IDH1* or *IDH2* mutations are associated with globally increased DNA methylation^15^,^17^, *MLL* fusions or mutations in *NPM1*, *DNMT3A* or *FLT3* were associated with decreased DNA methylation^15^.

Epigenetic signatures distinguishing normal haematopoietic stem and progenitor cells (HSPCs) are useful markers for survival analysis in AML^18^. However, these epigenetic changes have not previously been linked functionally to AML engraftment potential (that is, the LSC phenotype), and have only been investigated in mixed cell populations. Here we have attempted to define epigenetic differences between LSC and their non-stem blast progeny by testing engraftment capability of fractionated leukaemic cells from AML patients, to define the critical core elements of this key malignant stem cell phenotype. Through this approach, we define a methylation and gene expression-based epigenetic signature for LSC, and find it to be largely independent of genetic mutations and strongly implicating a role for the *HOXA* gene cluster. Finally, comparison of LSC with normal HSPC suggests that both L-MPP and GMP can give rise to LSC.

## Results

### AML LSC versus blasts define an LSC epigenetic signature

To formally investigate epigenetic differences between LSC and blast progeny, we sought to identify differentially methylated regions (DMRs) between functionally defined AML LSC-enriched populations and their downstream non-engrafting blasts from a cohort of 15 primary patient samples. We obtained samples from 15 AML patients (Supplementary Tables 1 and 2) and isolated subpopulations based on the expression of CD34 and CD38 including: Lin−CD34+CD38−; Lin−CD34+CD38+; and Lin−CD34− (Supplementary Fig. 1). We then performed comprehensive genome-scale DNA methylation analysis using the Illumina Infinium HumanMethylation450 bead chip array. While AML LSC were originally described to be exclusively contained in the CD34+CD38− subpopulation, recent reports have indicated that leukaemia-initiating cells can also be detected in multiple compartments including both the CD34+CD38+ and CD34− subpopulations, although usually at lower frequencies^3^,^4^,^5^.

To identify LSC and blast populations, we conducted xenotransplantation assays on all three CD34/CD38 subpopulations from each of the 15 AML cases (Supplementary Table 3). Similar to other reports, leukaemic engraftment was observed from at least one subpopulation in 10 out of 15 AML patients. As expected, LSC activity markedly decreased following the immunophenotypic hierarchy with 64.3% of CD34+CD38−, 46.7% of CD34+CD38+ and 26.7% of CD34− subpopulations engrafting *in vivo* (Supplementary Table 3). To identify epigenetic markers of functional LSC, we performed DMR analysis between the 20 LSC-containing (engrafting) and 24 blast-containing (non-engrafting) fractions (hereafter termed ‘LSC' and ‘Blast', respectively). The analysis identified 3,030 DMRs, of which 91.4% were hypomethylated in LSC (Table 1 and Supplementary Data 1). These DMRs were further classified according to their global genomic location including: islands (regions with a GC content >50% and an observed/expected CpG ratio of more than 0.6), shores (regions within 2 kb of an island), shelves (regions 2–4 kb away from an island) and open sea (isolated CpG sites in the genome without a specific designation). These DMRs were nearly evenly distributed in CpG islands (27.8%) and open seas (29%) (Table 1). In addition, the DMRs correlated with gene expression at CpG islands and open seas (Pearson's *r*=−0.12 (*P*=0.004) and −0.27 (*P*=0.003), respectively), whereas most hypomethylated DMRs in the engrafting populations were associated with transcriptional upregulation of associated genes (Supplementary Fig. 2).

We next sought to integrate DNA methylation with gene expression analysis to identify an LSC epigenetic signature by extracting genes, which passed a DMR *P* value <0.01 cutoff (permutation test) and exhibited >0.5 log_2_ ratio of differential expression between the LSC and Blast populations, with an inverse relationship between gene expression and DNA methylation within 2 kb of the transcriptional start site. We excluded gene body DMRs, as there was no statistically significant positive correlation in AML or normal haematopoiesis comparisons (Supplementary Fig. 3). We applied a minimum absolute value of log_2_ ratio of 0.5 differential expression, similarly to our previous LSC gene expression signature using the same microarray platform^6^. With these parameters, we identified 84 regions of 71 unique genes exhibiting differential methylation and gene expression in LSC compared with Blasts (Supplementary Data 2).

**Figure 1:**
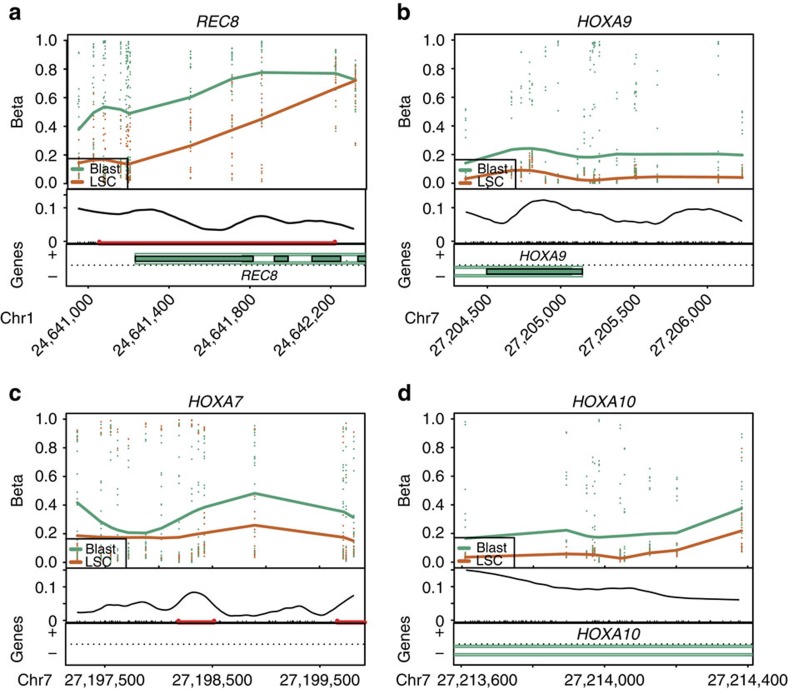
AML LSCs and Blasts exhibit epigenetic differences that define an LSC epigenetic signature. Plots of DMRs indicating genomic loci for *REC8* (**a**), *HOXA9* (**b**), *HOXA7* (**c**) and *HOXA10* (**d**), four LSC epigenetic signature genes that are hypomethylated and upregulated in LSC. Top: level of CpG methylation (Beta) of each sample for the region; middle: CpG density (curve), CpG sites (black tick marks) and CpG islands (red lines); bottom: gene annotation.

We compared our LSC epigenetic signature with the LSC gene expression signatures from previous studies^5^,^6^. Only six out of 71 genes were found in these earlier studies, suggesting most of the genes identified here comprise a novel signature for LSC defined first by DMR analysis and refined by gene expression differences. One gene in this signature, *REC8*, which encodes a kleisin family protein that is associated with the cohesin complex, was hypomethylated and transcriptionally upregulated in LSC (Fig. 1a and Supplementary Data 2). Notably, mutations of components of the cohesin complex have been identified in AML and other tumour types^19^,^20^. Further experiments will be necessary to determine if increased expression of *REC8* alters cohesin complex functions in LSC. We also identified *HOXA5*, *HOXA6*, *HOXA7*, *HOXA9* and *HOXA10* in the LSC epigenetic signature (Fig. 1b–d and Supplementary Data 2). These *HOXA* cluster genes were hypomethylated and highly expressed in LSC (Supplementary Data 2). Notably, *HOXA9* showed hypomethylation and increased expression in LSC (Fig. 1b and Supplementary Data 2), and aberrant expression of *HOXA9* is known to be involved in increased proliferation of HSPCs and leukaemogenesis, suggesting a critical role in LSC activity^21^,^22^,^23^,^24^.

Because the mixed-lineage leukaemia (MLL) subtype is itself associated with changes in expression of members of the *HOXA* gene cluster^25^,^26^, we performed a second DMR and gene expression analysis excluding the five LSC populations from the two MLL patients in our cohort to obtain an LSC epigenetic signature without MLL cases. We observed substantial overlap between the sets of DMRs without MLL cases and with all samples. For the key LSC epigenetic signature 81 of 84 DMRs were present after removal of the MLL cases (Supplementary Table 4). Considering all DMRs with *P*<0.01 (not just the LSC signature), there was 77% overlap (Supplementary Table 4). The LSC epigenetic signature without MLL cases showed substantial overlap including *HOXA* genes (Supplementary Table 4). Note that the reduction of DMRs within the LSC epigenetic signature without MLL cases is primarily due to decreased sample numbers of LSC, which reduced statistical power as 5 samples out of 20 LSC were excluded. To clarify this point, we performed an enrichment test to see if the 1,632 DMRs, which were excluded after MLL cases were removed, were still enriched among the DMRs without MLL cases with less strict statistical cutoff (*P* <0.05 rather than *P*<0.01, permutation test). We observed statistically significant enrichment of these excluded DMRs in the DMRs without MLL cases (*P*<2.2 × 10^−16^, *χ*^2^-test). In addition, we performed an enrichment test to examine if the LSC epigenetic signature without MLL cases was enriched in the LSC epigenetic signature with all samples. We observed statistically significant enrichment between those two LSC epigenetic signatures (*P*<2.2 × 10^−16^, *χ*^2^-test). Thus, the presence of the MLL subtype was not a confounding variable in defining the LSC epigenetic signature with the inclusion of the *HOXA* cluster.

### The LSC epigenetic signature is largely mutation independent

To identify the important pathways and upstream regulators of LSC activity, we utilized Ingenuity Pathway Analysis. The most significantly enriched pathway was fatty acid α oxidation (Supplementary Data 3), and inhibitors of this pathway have been previously shown to induce apoptosis of leukaemia cells^27^. Ingenuity upstream regulator analysis identified *NPM1*, *ASXL1* and *KAT6A* as the most significant upstream regulators of the LSC epigenetic signature genes, primarily through regulation of *HOXA* genes including *HOXA5*, *HOXA6*, *HOXA7*, *HOXA9* and *HOXA10* (Supplementary Data 4). Significantly, all three of these upstream regulators have been found to be mutated in AML and likely serve as driver genes^15^. In particular, mutations in *ASXL1* and *NPM1* have been shown to cooperate with HOX genes to initiate leukaemia by enhancing self-renewal and proliferation of haematopoietic progenitors^16^,^28^. Consistent with this, we observed that *NPM1* mutation was associated with decreased methylation and increased expression of *HOXA5*, *HOXA6*, *HOXA7*, *HOXA9* and *HOXA10* compared with *NPM1* wild-type samples in the The Cancer Genome Atlas (TCGA) cohort (Supplementary Fig. 4).

**Figure 2:**
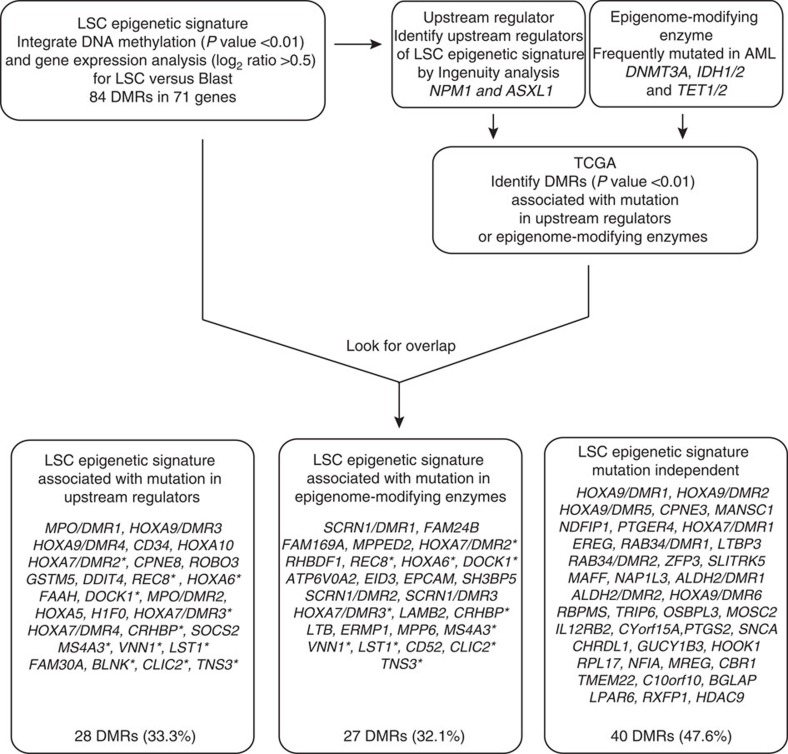
The LSC epigenetic signature is partially dependent on underlying somatic mutations. Shown is a schematic flow chart of mutation association analysis. We compared our LSC epigenetic signature with the mutation-specific DMRs obtained from TCGA data set. The LSC epigenetic signature was classified into three different groups, and each DMR is shown in this diagram. Note that several genes such as *HOXA9* have multiple DMRs and different DMRs in one gene are annotated with DMR number such as *HOXA9/DMR1*. Genes associated with mutation in both upstream regulators and epigenome modifying enzymes are marked with `*'.

**Figure 3:**
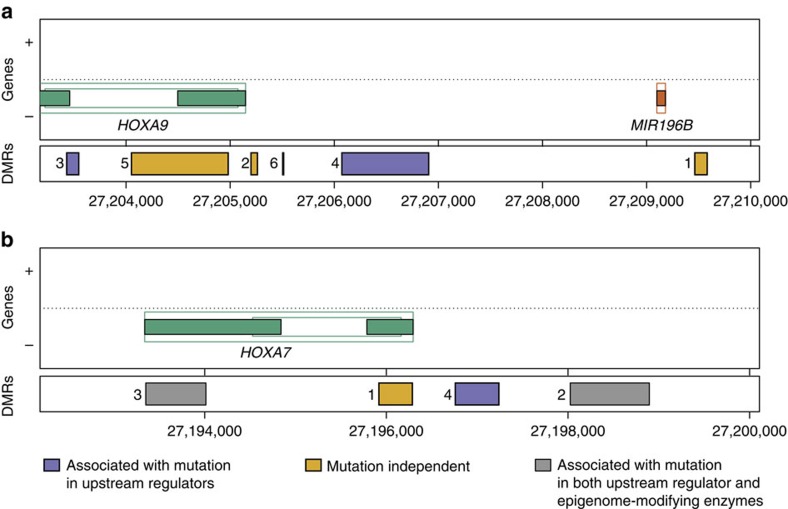
Examples of multiple DMRs for a gene. Top panel shows gene annotation and bottom panel shows genomic location of multiple DMRs with a number on the left, corresponding to the DMR number in Supplementary Data 5. DMRs are coloured differently according to the classification in Fig. 2, as indicated at the bottom. (**a**) *HOXA9*. (**b**) *HOXA7*.

We then sought to investigate the LSC epigenetic signature for its association with AML mutations in the TCGA cohort^15^ (Fig. 2). The TCGA cohort consists of 200 AML patient samples with associated DNA methylation, gene expression and full genotyping from genome/exome sequencing^15^. First, we identified the epigenetic signatures associated with individual AML mutations by performing DMR analysis between wild-type and mutant patient samples (Fig. 2). The mutations tested included epigenome-modifying enzymes such as *DNMT3A*, *IDH1/2* and *TET1/2*, and upstream regulators of our LSC epigenetic signature, *NPM1* and *ASXL1* (Fig. 2). *KAT6A* was not included as there was no patient who had this mutation among the patients investigated on methylation arrays. Next, we examined the overlap between the mutation-associated DMRs and our LSC epigenetic signature (Fig. 2). Each LSC epigenetic signature gene was classified into three categories: (1) upstream regulator-associated if differentially methylated in association with any mutation in upstream regulators; (2) epigenome-modifying enzyme-associated if differentially methylated in association with any mutation in epigenetic enzymes; or (3) mutation independent if it was not differentially methylated in association with either upstream regulator or epigenome-modifying enzyme (Fig. 2 and Supplementary Data 5). Of the 84 LSC DMRs, 28 (33.3%) and 27 (32.1%) were associated with upstream regulator or epigenome-modifying enzyme mutations, respectively (Fig. 2 and Supplementary Data 5). However, 40 DMRs (47.6%) including *HOXA7* and *HOXA9* were mutation-independent targets (Fig. 2 and Supplementary Data 5). It should be noted that some of the LSC differentially methylated genes, including *HOXA7* and *HOXA9*, have multiple DMRs regulated by different mechanisms (Fig. 3). For example, *HOXA7* has four DMRs in the LSC epigenetic signature; one associated with mutation in *NPM1*, two associated with mutation in *DNMT3A*, *TET1* and *NPM1*, and one mutation independent (Supplementary Data 5). Therefore, we annotated each DMR in those genes differently with DMR numbering such as *HOXA9/DMR1* (Figs 2 and 3, and Supplementary Data 5). A small subset (11 signatures) of upstream regulator and epigenetic enzyme-associated LSC epigenetic signatures overlapped, including *REC8*, *HOXA6* and *HOXA7* (Fig. 2 and Supplementary Data 5). This analysis showed that all the *HOXA* genes are epigenetically regulated by at least one upstream regulator, and *HOXA6*, *HOXA7/DMR2* and *HOXA7/DMR3* are common targets of both upstream regulators and epigenetic enzymes (Figs 2 and 3, and Supplementary Data 5), and all of these changes involved DNA hypomethylation. In addition, hypomethylation of *HOXA7/DMR1* occurred independently of mutations (Figs 2 and 3, and Supplementary Data 5). Together, these results suggest that overexpression of *HOXA* genes mediated by DNA hypomethylation is a core mechanism for LSC activity.

### The LSC epigenetic signature is prognostic in human AML

**Figure 4:**
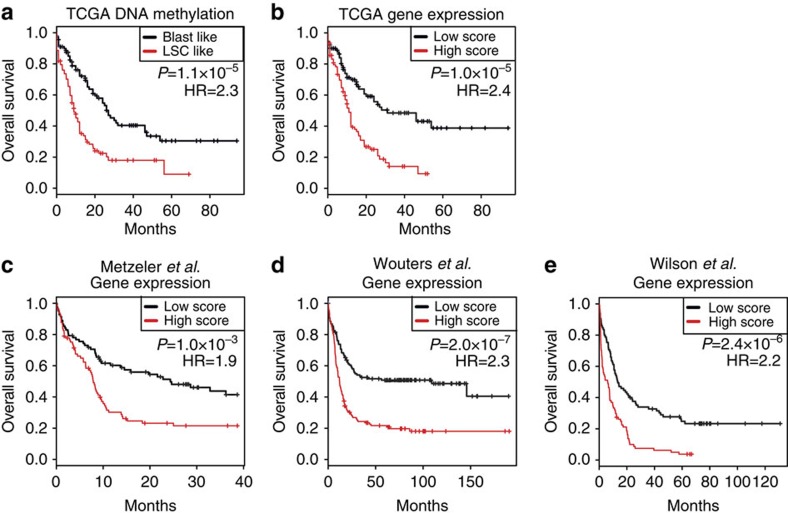
The LSC epigenetic signature is associated with overall survival in human AML. (**a**) TCGA samples were classified as LSC like or Blast like based on DNA methylation alone by generating methylation profiles of the LSC and Blast populations, and then calculating scores of each sample based on the probability of being closer to either LSC or Blast. Kaplan–Meier survival analysis was then applied to these groups as indicated. Statistical significance was determined by the log-rank test (*n*=192; 93 LSC-like and 99 Blast-like patients). (**b**–**e**) Expression of the LSC epigenetic signature genes was combined to create an LSC score, which was then calculated in AML samples. The first principal component of genes in the LSC signature was computed, and patients were stratified as ‘high' or ‘low' relative to its median value in four independent cohorts including TCGA (*n*=182; 91 high-score and 91 low-score patients), (**b**) Metzeler *et al*. (*n*=163; 81 high-score and 82 low-score patients) (**c**), Wouters *et al*. (*n*=262; 131 high-score and 131 low-score patients) (**d**) and Wilson *et al*. (*n*=169, 84 high-score and 85 low-score patients) (**e**). In each cohort, patients were classified into high and low groups based on the median value. Kaplan–Meier survival analysis was then applied to these groups as indicated. Statistical significance was determined by the log-rank test.

We hypothesized that if the LSC epigenetic signature reflected key drivers of the functional differences between LSC and Blasts, then this signature should be associated with clinical outcomes in human AML. First, we tested the association between the LSC epigenetic signature and overall survival in the DNA methylation data from the TCGA AML cohort^15^. To assign each TCGA patient to an LSC-like or Blast-like category, we calculated scores of each TCGA sample based on the probability of being closer to either LSC or Blasts. A comparable number of samples were assigned to each category by this method (99 for Blast like and 93 for LSC like). In univariate survival analysis, the LSC-like group showed worse outcome compared with the Blast-like group (hazard ratio (HR)=2.3, 95% confidence interval (CI)=1.6–3.4; *P*=1.1 × 10^−5^, log-rank test; Fig. 4a). The LSC-like versus Blast-like stratification remained associated with overall survival in multivariate analysis together with other known prognostic factors such as age (considered as a continuous variable), cytogenetic risk (assessed as high versus low risk and intermediate versus low risk), *NPM1* and *FLT3* mutations (HR=1.9, 95% CI=1.2–2.9; *P*=0.003, log-rank test; Table 2).

Next, we tested the association between expression of LSC epigenetic signature genes and clinical outcome using four different cohorts including TCGA^15^, a cohort of normal karyotype patients^29^,^30^, and two cohorts of mixed karyotype patients^31^,^32^,^33^. These cohorts consist of a total of 776 AML patients treated on different clinical protocols that also exhibited distinct biological characteristics^6^. We observed a strong correlation between the relative expression of LSC epigenetic signature genes and overall survival in the TCGA cohort (correlation=0.49; *P*=4 × 10^−13^; Supplementary Fig. 5). The more highly expressed a gene was in LSC compared with Blasts, the more robust its association with worse overall survival. In all four cohorts, the overall expression level of the signature genes was significantly associated with overall survival, with higher expression associated with worse clinical outcomes in the TCGA cohort (HR=2.4, 95% CI, 1.6–3.6; *P*=1x10^−5^, log-rank test; Fig. 4b, Supplementary Table 5). This association remained significant in multivariate Cox regression including age (continuous), cytogenetic risk, *NPM1* and *FLT3* mutations (HR=1.7, 95% CI, 1.0–2.7); *P*=0.03, log-rank test; Table 2). Similar results were observed for the three other cohorts in univariate and multivariate analyses (Fig. 4c–e, Supplementary Table 5 and Table 3).

Finally, we tested if mutations in epigenetic enzymes such as *DNMT3A*, *IDH1/2*, *TET2* and *ASXL1* affected the prognostic impact of the LSC epigenetic signature in the TCGA cohort. As described previously, mutation in *DNMT3A*, but none of the other genes, was associated with patient overall survival (Supplementary Table 6). Multivariate survival analysis including *DNMT3A* mutation showed that our LSC epigenetic signature remained independently associated with clinical outcome in both the DNA methylation and gene expression data from TCGA, even when incorporating cytogenetic risk group (Supplementary Table 7), as well as within the intermediate cytogenetic risk group alone (Supplementary Table 8). Overall, these results demonstrate that the LSC epigenetic signature defined by DNA methylation and gene expression is associated with overall survival in human AML.

### Epigenetically distinct LSC reflecting the cell of origin

To address the question of the cell of origin of AML LSC, we first analysed normal haematopoiesis that proceeds through a series of multipotent and oligopotent stem and progenitor cells that progressively lose self-renewal ability and become more restricted in their differentiation potential (Supplementary Fig. 6a). We reasoned that comparison of DNA methylation profiles of AML populations to those of normal HSPC would imply the cell of origin of AML LSC. We obtained bone marrow from five normal donors and isolated HSPC by fluorescence-activated cell sorting (FACS) including: HSC, multipotent progenitors (MPP), L-MPP, CMP, megakaryocyte/erythroid progenitors (MEP) and GMP (Supplementary Fig. 6b and Supplementary Tables 9 and 10).

**Figure 5:**
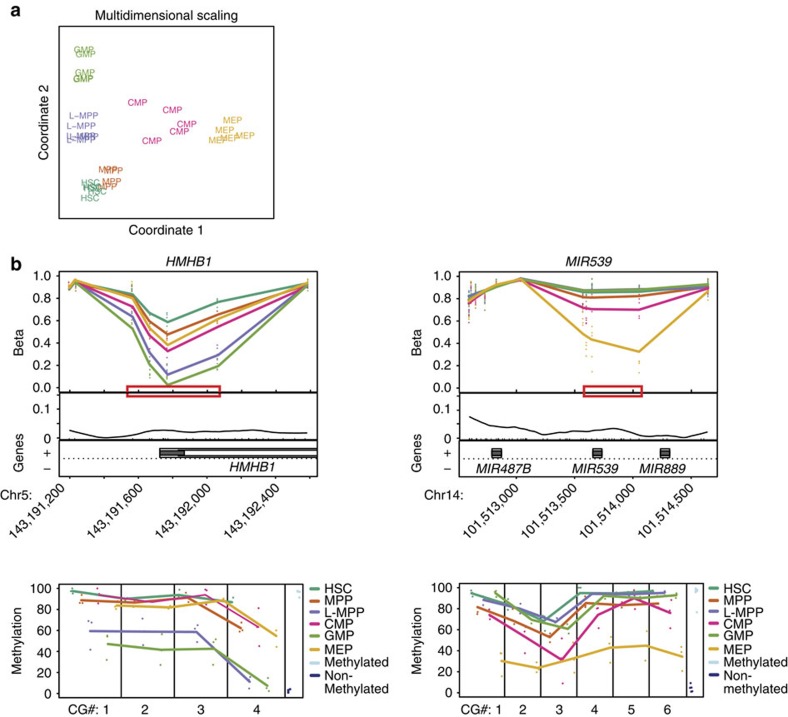
Comprehensive DNA methylation analysis shows tight clustering of individual HSPC populations. (**a**) Multidimensional scaling examining the top 1,000 most variable methylation positions among HSPC populations shows tight clustering of distinct lineages. (**b**) DMR plots show genomic loci for newly identified genes with previously unknown functions in haematopoiesis. The examples are *HMHB1* and *MIR539*. Top: level of CpG methylation (Beta) of each sample for the region; middle: CpG density (curve), CpG sites (black tick marks) and CpG islands (red lines); bottom: gene annotation; lower panel: bisulfite pyrosequencing replicating the methylation value for individual CpGs in the red boxes.

To further understand epigenetic variation during early human haematopoiesis, we generated genome-scale methylation profiles for these normal HSPC and subjected them to DMR analysis. Multidimensional scaling analysis utilizing the top 1,000 most variable CpG positions revealed tight clustering of human HSPC populations by lineage with no outliers (Fig. 5a). This analysis indicates that DNA methylation reflects the identity of the HSPC populations.

The DMRs identified across HSPC potentially reveal novel regulators of haematopoietic lineage development by identifying previously unknown sites of epigenetic variation during haematopoiesis (Fig. 5b and Supplementary Data 1). For example, *HMHB1*, encoding one of the minor histocompatibility antigens, was found to be hypomethylated in L-MPP and GMP, suggesting a possible role in GMP differentiation (Fig. 5b). Progressive hypomethylation was also identified in *MIR539* going from HSC to MEP, suggesting that this microRNA may contribute to erythropoiesis (Fig. 5b). Interestingly, the *MIR539* gene is located in the DLK1-DIO3 imprinting region that contains a microRNA cluster involved in leukaemia pathogenesis^34^. Further validation of these novel candidate regulators will require functional experiments. Genes with an inverse correlation between DNA methylation and gene expression were located outside of the islands themselves, with the strongest correlation at shores and open seas for HSC/GMP and HSC/MEP comparisons (Supplementary Fig. 7). In addition to these comparisons, more than 50% of the DMRs among HSPCs were in open seas (Table 1). Thus, functional epigenetic differences during early human haematopoietic differentiation occur in CpG sparse regions, consistent with other recent studies of differentiation^35^,^36^ and cancer^36^,^37^.

**Figure 6:**
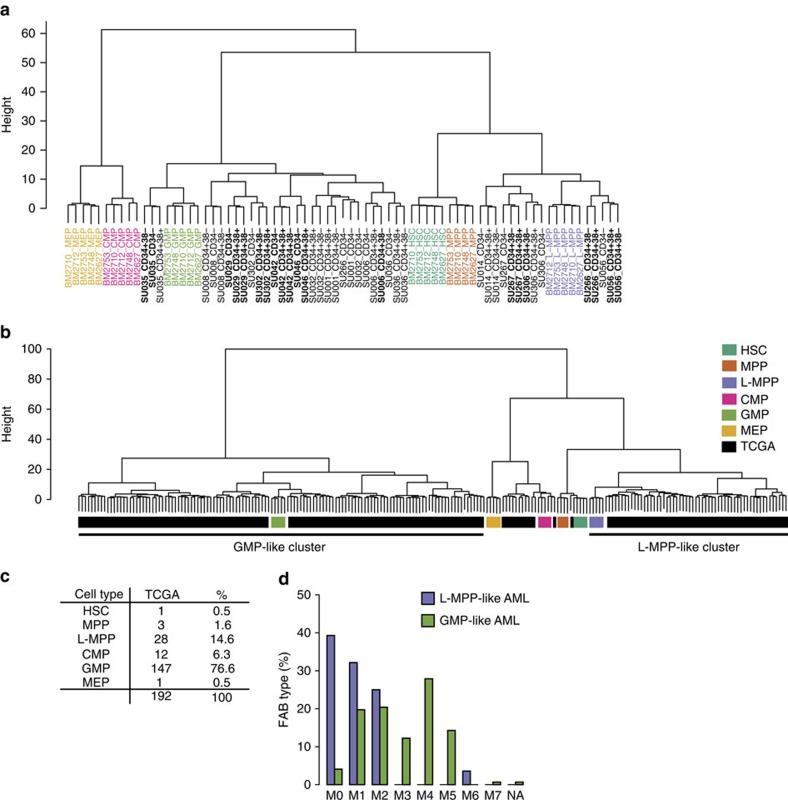
Epigenetic signatures define subgroups of AML LSC reflecting the cell of origin. (**a**) A total of 216 DMRs identified from all possible pairwise comparisons among six HSPCs were used to cluster all normal HSPCs with all AML subpopulations. The primary AML subpopulations form two major clusters: L-MPP like and GMP like. LSC subpopulations are indicated in bold. (**b**) Clustering analysis of TCGA AML samples with normal human HSPC using the 216 DMRs shows that the L-MPP-like and GMP-like clusters are observed in this cohort as well. (**c**) TCGA samples were classified according to normal HSPC populations based on DNA methylation alone by generating methylation profiles of all the normal HSPC, and then calculating scores of each sample based on the closest population. The normal progenitor cell identity for all the TCGA samples is indicated. (**d**) The L-MPP-like and GMP-like TCGA samples were grouped according to their FAB classification. NA, not classified.

To relate normal haematopoiesis to LSC, we first identified the DMRs from all possible pairwise comparisons among the six HSPCs after applying a more rigorous cutoff of family-wise error rate <0.1 (Supplementary Data 6). The resulting 216 DMRs were applied in clustering analysis including all six normal HSPC populations with LSC and Blasts (Fig. 6a). Strikingly, this analysis revealed that AML samples formed two distinct clusters, L-MPP like and GMP like (Fig. 6a). Importantly, the GMP-like cluster included several CD34+CD38− subpopulations, indicating that these clusters could not have been identified by immunophenotype alone. Moreover, clustering analysis using an equal number of length-matched random regions showed that the clustering of AML populations with either L-MPP or GMP was unique to the selected DMRs (Supplementary Fig. 8).

Strikingly, using the same 216 DMRs, the TCGA samples also formed the same two major clusters, L-MPP like and GMP like (Fig. 6b). In addition to the two major clusters, we also identified a minor CMP-like cluster that was not observed in our smaller cohort. We calculated scores indicating the similarity of each TCGA sample to each of the six progenitors, and designated a counterpart HSPC population for each TCGA sample based on highest similarity. This approach showed that 76.6% of TCGA samples resembled GMP and 14.6% had a methylation profile most similar to L-MPP (Fig. 6c).

We hypothesized that if the assignment of AML samples to L-MPP-like and GMP-like clusters was related to the cell of origin, then the degree of maturity and morphology might differ between the two groups. Consistent with this, we compared the distribution of the French–American–British (FAB) classification of the TCGA samples and found that the L-MPP-like cases mainly consisted of more immature M0, M1 and M2 types, while the more differentiated M4 and M5 types were enriched in GMP-like AML (*P*<1 × 10^−4^, *χ*^2^-test, Fig. 6d). It should be noted that the LSC epigenetic signature is not merely a recapitulation of FAB types, as our signature is prognostic in multivariate analysis while FAB types are not (Supplementary Table 11). In addition, it is not possible to know the cell of origin simply by examining FAB types (Supplementary Table 12). For example, in 38 cases of M1 AML, 29/38 are GMP like and 9/29 are L-MPP like (Supplementary Table 12).

Finally, we sought to investigate if the L-MPP-like and GMP-like clusters, and therefore the potential cell of origin, were associated with cytogenetic abnormalities or recurrent mutations of specific genes including *DNMT3A*, *IDH1*, *IDH2*, *TET1*, *TET2*, *FLT3* and *NPM1*. The GMP-like cluster was enriched for patients in the low- and intermediate-cytogenetic-risk groups, while the L-MPP-like cluster was enriched for patients in the high-cytogenetic-risk group (*P*=1x10^−4^, Fisher's exact test; Supplementary Table 13). We found that *IDH1* and *IDH2* mutations were enriched in the L-MPP-like group (*P*<0.01 for both, Fisher's exact test), and *FLT3* and *NPM1* mutations were enriched in the GMP-like group (*P*<0.01 for both, Fisher's exact test). *DNMT3A* and *TET1* mutations were more enriched in the L-MPP group, but this was not statistically significant (Supplementary Table 14). Together, these results demonstrate that DNA methylation signatures permit a novel clustering of AML into L-MPP-like and GMP-like groups that may reflect the cell of origin for each case and demonstrate an association with key disease features. Ultimately, these results are suggestive of the cell of origin in AML, but definitive proof is not possible in any human primary cancer.

## Discussion

The cancer stem cell model was originally proposed based on observations from human AML in which only subpopulations of leukaemia-initiating or LSC possessed engraftment potential^38^,^39^. According to this model, the LSCs give rise to downstream Blasts that lack critical stem cell properties. As LSCs and their non-engrafting Blast progeny are clonally related, a major implication of this leukaemia stem cell model is that their functional properties must be due to epigenetic differences. Here we provide such evidence by characterizing global DNA methylation features of LSC defined by xenotransplantation of AML subpopulations, compared with non-engrafting Blast cells, demonstrating that AML LSCs exhibit global hypomethylation compared with non-LSC Blast cells. Integrating DNA methylation and gene expression analysis, we identified 84 regions of 71 genes as the LSC epigenetic signature. A total of 65 of these 71 genes were not reported in previous gene expression studies for LSC (the exceptions being *CD34*, *SH3BP5*, *RBPMS*, *LTB*, *MS4A3* and *VNN1* (refs 5, 6)). Most of the LSC epigenetic signature was mutation independent, not associated with mutations in upstream regulators or epigenome-modifying enzymes suggesting that leukaemogenesis may converge on these primary epigenetic signatures. We also identified some mutation-associated epigenetically dysregulated genes, including *REC8* and *HOXA7*. Together, these epigenetic signatures represent potential therapeutic targets regardless of the different types of the underlying mutations present in individual AML cases. Furthermore, the LSC epigenetic signature was prognostic of patient overall survival independently of known survival predictors such as age and cytogenetic abnormalities, emphasizing its functional importance. Finally, by mapping the epigenome of early normal haematopoiesis, we also determined that epigenetic signatures define clusters of GMP-like and L-MPP-like AML LSCs that likely reflect the cell of origin and are associated with key clinical features including cytogenetic abnormalities and molecular mutations.

Apart from its prognostic effect, the LSC epigenetic signature represents a molecular target that may improve patient survival and prevent relapse. Recently, epigenetic therapy with hypomethylating agents such as azacytidine and decitabine has been approved for the treatment of AML. Randomized trials demonstrated improved overall survival compared with chemotherapy, but also indicated limited effect on relapse rate in high-risk AML^40^. Our results indicate that LSCs are relatively hypomethylated compared with Blasts, suggesting that they may be less effectively targeted by hypomethylating agents, possibly accounting for their limited efficacy in relapse-free survival. It would be of great interest to see how the LSC epigenetic signature is affected by these drugs.

More specifically, this LSC epigenetic signature was markedly enriched for the members of the *HOXA* cluster, suggesting this cluster is a key driver of LSC function. The *HOXA* cluster has been implicated as a key regulator of haematopoiesis and myeloid malignancy^41^. In particular, *HOXA9* is known to be involved in increased proliferation of HSPC and leukaemogenesis^21^, even occurring as a fusion oncogene in rare cases^42^. Moreover, increased expression of *HOXA9* has been found to be an adverse prognostic factor in AML^43^. Other *HOXA* family members including posterior (*HOXA7* and *HOXA10*) and anterior (*HOXA6*) members have been implicated in leukaemogenesis, as overexpression of these genes in normal mouse HSPC leads to increased self-renewal, transformation and development of myeloid malignancies^44^. The functional LSC epigenetic signature provided here demonstrates that the *HOXA* family is a key driver of AML LSC that may function in imparting aberrant self-renewal.

As DNA methylation is a potential marker of cell identity, here we compared DNA methylomes of normal HSPC with LSC as a marker for the cell of origin in AML. Using this approach, we observed two subtypes in our cohort: L-MPP-like and GMP-like. These two subtypes were also identified in the TCGA cohort, suggesting that leukaemic transformation predominantly occurs at either the L-MPP or GMP stage of haematopoietic development. We found that other features of AML were associated with these two subtypes including FAB type, several mutations and cytogenetic abnormalities, suggesting that the cell of origin may drive key clinical features in AML.

We newly identified a small subset of AML cells clustering with CMP and few samples clustering with HSC, MPP and MEP that could not be identified in smaller data sets including our own data and a previous study^3^. The result from TCGA indicates that the cell of origin of AML could be variable among HSPC. The result for the cell of origin should be considered carefully, as it is hard to provide definite proof of the question due to experimental limitations. Despite this caveat that affects all of human primary cancer biology, we do provide the first epigenetic evidence for cell of origin in human leukaemia and believe that our approach using epigenomic profiles suggests an efficient way to study cell of origin in cancer biology using large data sets.

## Methods

### Human samples

Fresh human bone marrow mononuclear cells (BMMCs) from healthy donors (2 × 10^8^ cells per donor, Catalogue#: ABM006) were purchased from ALLCELLS (Emeryville, CA). A CD34+ cell-enrichment step was performed with the human progenitor cell-enrichment kit with CD61 depletion (Stem Cell Technologies, Canada, Catalogue# 19356) on a RobSep machine from the same company. Human AML samples were collected from patient peripheral blood or bone marrow at Stanford Hospital, according to a Stanford-IRB-approved protocol (22264), and informed consent was obtained from all subjects. Peripheral blood mononuclear cells (PBMCs) or BMMCs were separated with Ficoll-Paque Plus (Amersham Biosciences, Piscataway, NJ, Catalogue number: 17-1440-03), and cryopreserved in 1 × freezing medium (90% fetal bovine serum (FBS)+10% dimethylsulfoxide). All the AML experiments were conducted with cryopreserved PBMC or BMMC samples that were thawed and washed in IMDM medium containing 10% FBS.

### TCGA patient sample data

DNA methylation, gene expression, somatic mutation and clinical information were derived from the TCGA Research Network: http://cancergenome.nih.gov/.

### Flow cytometry analysis and cell sorting

A battery of antibodies (Supplementary Table 10) was used for staining, analysis and sorting of progenitor cells from either healthy BMMCs or AML patient PBMCs/BMMCs, as well as lineage analysis human chimerism/engraftment. Cells were either analysed or sorted using a FACS Aria II cytometer (BD Biosciences, Franklin Lakes, NJ). Analysis of flow cytometry raw data was done with FlowJo Software (Treestar, Ashland, OR).

### Xenotransplantation assay

NOD.Cg-*Prkdc*^*scid*^ *Il2rg*^*tm1Wjl*^/SzJ mice (NSG) were obtained from The Jackson Laboratory (Bar Harbor, ME) and bred in a specific pathogen-free environment per Stanford Administrative Panel on Laboratory Animal Care Guidelines (Protocol 22264). Six- to eight-week-old female adult mice were exposed to 200 rad of gamma irradiation at least 2 h (up to 24 h) before transplantation. Up to 500 thousand fresh-sorted AML cell subpopulation were resuspended in 30 μl of Hank's balanced salt solution (Gibco Life Technologies, Grand Island, NY) containing 2% FBS, and injected intravenously via the tail vein using a 29-gauge needle. For each cell subpopulation, at least three technical replicates were performed by transplantation of three aliquot of cells into three mice. It was counted as ‘Yes' if at least one of the three mice was engrafted (Supplementary Table 3). Around 150 mice in total were used. Neither randomization nor blinding was used for this study.

After 8 weeks, mice were euthanized with CO_2_ according to Stanford-IRB-approved protocol (22264). Bone marrows were isolated using scissors and needle flashing, then underwent hypotonic red cell lysis using ACK (ammonium–chloride–potassium) lysing buffer (Life Technologies, Grand Island, NY, Catalogue# A10492). BMMCs were stained with antibody combinations (Supplementary Table 10) on ice for 30 min, and dead cells were excluded by propidium iodide staining. Human myeloid engraftment (hCD45+CD33+) and lymphoid engraftment (hCD45+CD19+) were analysed on flow as described before.

### Illumina infinium human methylation 450 bead array assay

Genomic DNA from each sample was purified using the MasterPure DNA purification kit (Epicentre) according to the manufacturer's protocol. The genomic DNA (250–500 ng) was treated with sodium bisulfate using the Zymo EZ DNA Methylation Kit (ZYMO Research) as recommended by the manufacturer, with the alternative incubation conditions for the Illumina Infinium Methylation Assay. Converted DNA was eluted in 11 μl of elution buffer. DNA methylation level was measured using Illumina Infinium HD Methylation Assay (Illumina) according to the manufacturer's specifications. Methylation array data are deposited at the Gene Expression Omnibus (GEO) with accession number GSE63409.

### Illumina infinium human methylation 450 bead array analysis

Raw intensity files were obtained using minfi package^45^ to calculate methylation ratios (Beta values). The data was normalized using Illumina preprocessing method implemented in minfi. Several quality control measures were applied to remove arrays with low quality. Control probes were examined on the 450k array to assess several measures including bisulfite conversion, extension, hybridization, specificity and others. One of the MPP samples (BM2712) and two samples of TCGA (patient IDs: 2934 and 2827) showed low quality for the measures, so they were removed for further analysis. Next, median methylated and unmethylated signals were calculated for each arrays; no array was identified for signal values lower than 10.5. For multidimensional scaling analysis, probes containing an annotated single-nucleotide polymorphism (SNP) (dbSNP137) at the single-base extension or CpG sites were removed (17,398 probes removed). Minfi 1.8.9 was used.

Bump hunting method previously described was applied to identify DMRs in 450k array^45^,^46^. Beta value of 0.1 (10% of methylation difference) was used as cutoff when finding DMRs. Statistical significance was assigned by permutation testing and the *P* value cutoff used for downstream analysis was <0.01 that corresponded to Benjamini–Hochberg adjusted *P* value <0.1 (data not shown) unless different cutoff was designated in result part. Bumphunter 1.2.0 was used. Same method was applied to identify DMRs for the second DMR analysis of LSC versus Blast that we removed five LSC cases from two MLL patients (SU042 and SU046).

### Bisulfite pyrosequencing

A unit of 100 ng of genomic DNA from each sample was treated with sodium bisulfate using an EZ DNA Methylation Gold Kit (ZYMO Research) following the manufacturer's protocol. The bisulfate-treated DNA was PCR amplified using unbiased nested primers. Quantitative pyrosequencing was performed using a PSQ HS96 (Biotage) to validate DMR regions. The DNA methylation percentage at each CpG site was measured using the Q-CpG methylation software (Biotage). SssI-treated human genomic DNA was used as 100% methylated controls and human genomic DNA amplified by Repli-G mini kit (Qiagen) was used as the non-methylated (0%) DNA control. Supplementary Table 15 provides the primer sequence used for the pyrosequencing reactions with the chromosomal coordinates in the University of California at Santa Cruz February 2009 human genome assembly (hg19) for each CpG site investigated.

### Affymetrix microarray expression analysis

Total RNA was extracted from each FACS-sorted cell population using RNeasy Plus Mini (QIAGEN, Valencia, CA, Catalogue#: 74134) according to the manufacture's protocol. All RNA samples were quantified with 2100 Bioanalyzer (Agilent Technologies, Santa Clara, CA), subjected to reverse transcription, two consecutive rounds of linear amplification, and production and fragmentation of biotinylated cRNA. A unit of 15 μg of cRNA from each sample was hybridized to HG U133 Plus 2.0 microarrays. Hybridization and scanning were performed according to the manufacture's instruction (Affymetrix). This step was performed at the PAN Center of Stanford University. Data were normalized by GC robust multi-array average method and analysed on R/Bioconductor. SU042 CD34+38+, BM2770 GMP, BM2759 L-MPP, BM2761 CMP and BM2770 CMP were removed from further analysis due to low quality. SU001 was excluded, as the samples from this patient were not included on expression array (GEO accession code: GSE63270).

### Sanger sequencing to detect AML mutations

Genomic DNA was extracted from patient BMMC or PBMC using QIAmp DNA Mini Kit (QIAGEN, Valencia, CA, Catalogue#: 51304) according to the manufacture's instruction. PCR primers were designed to cover exon 3–11 of TET2, exon 4 of IDH1/2 and exon 7–23 of DNMT3A (Supplementary Table 16). The PCR reaction premix consists of 1 × of OneTaq 2 × Master Mix (NEB, Ipswich, MA, Catalogue#: M0482L), 0.2 μM forward and reverse primers, respectively, and 10 ng (up to 100 ng) genomic DNA as template. The reaction was under the condition of 95 °C initial denaturation for 30 s, 45 cycles of extension containing 94 °C for 30 s, 56 °C for 1 min (or as necessary) and 72 °C for 1 min, and a final extension at 72 °C 5 min. The PCR products were concentrated with PCR purification kit (QIAGEN, Valencia, CA, Catalogue#: 28106), then submitted to Sequentech (Mountain View, CA) for sequencing of both forward and reverse directions using 3730xl DNA Analyzer (Applied Biosystems, Foster City, CA) according to the manufacturer's instruction. The sequencing data were analysed using Sequencher 5.1 (Gene Codes Corporation, Ann Arbor, MI), and SNP was excluded by checking NCBI website before getting the final mutation results.

### Survival analysis

Survival analysis was performed to assess the association of LSC DNA methylation and gene expression signatures with clinical outcome (overall survival) in four different cohorts. For DNA methylation data set (TCGA), patients were separated into two groups; LSC like and Blast like based on the methylation profile of each individual. Survival was compared between the two groups using the *coxph* function in R (survival package 2.37), with significance assessed by log-rank test. For gene expression, the genes in the LSC epigenetic signature were identified in expression data sets for which survival outcomes were available. The first principal component of their expression levels was computed, and patients were stratified as ‘high' or ‘low' relative to its median value. Survival differences between the groups were assessed by log-rank test. In multivariate analyses, age was incorporated as a continuous variable, mutations were coded as present/absent (1/0) and assessment of cytogenetic risk was treated as individual groups and done for intermediate versus low risk and high versus low risk (Supplementary Software 1). Analysis was also performed within intermediate-risk groups.

### Statistical analysis

To assign cell identity of LSC/Blast to TCGA samples, mean methylation value of each LSC epigenetic signature (84 DMRs) for LSC/Blast (methylation profile) was retrieved and s.d. of the mean value for each signature was calculated. Then scores (probability density values as log value) for each TCGA sample regarding LSC and Blast profile was calculated using dnorm function with the mean and s.d. calculated in previous step. Maximum value of scores between the ones regarding LSC and Blast methylation profile was chosen, and then cell identity assigned. Same strategy was applied using the 216 DMRs for normal haematopoiesis to assign normal progenitor identity to TCGA samples in Fig. 6c. R code is available on request.

For clustering analysis, hclust function with ward method in R was used to generate all the cluster dendrogram analysis.

The test to examine the enrichment of the excluded DMRs after removing MLL cases in DMRs without MLL cases using a less strict statistical cutoff (*P*<0.05) was a *χ*^2^-test based on the number of overlapping DMRs between the excluded DMRs after removing MLL cases and DMRs without MLL cases (*P*<0.05), then DMRs only in the excluded DMRs and DMRs only in DMRs without MLL cases (*P*<0.05), and finally random DMRs from array background that do not overlap with either the excluded DMRs or DMRs without MLL cases (*P*<0.05).

### Bioinformatics analysis

QIAGEN's Ingenuity Pathway Analysis (Ingenuity Systems, www.ingenuity.com) was performed for pathway analysis.

## Additional information

**Accession codes:** Methylation array data and microarray data have been deposited in the Gene Expression Omnibus (GEO) under accession codes GSE63409 and GSE63270, respectively.

**How to cite this article:** Jung, N. *et al*. An LSC epigenetic signature is largely mutation independent and implicates the HOXA cluster in AML pathogenesis. *Nat. Commun.* 6:8489 doi: 10.1038/ncomms9489 (2015).

## Supplementary Material

Supplementary Information Supplementary Figures 1-8, Supplementary Tables 1-16 and Supplementary References

Supplementary Data 1 DMR lists for the comparisons in Table1

Supplementary Data 2 LSC epigenetic signature

Supplementary Data 3 Ingenuity pathway analysis

Supplementary Data 4 Ingenuity upstream regulator analysis

Supplementary Data 5 Association of LSC epigenetic signature with DMRs of genetic mutations

Supplementary Data 6 DMRs for normal hematopoiesis

Supplementary Software 1 R script for multivariate survival analysis

## Figures and Tables

**Table 1:** Summary of DMRs identified in the indicated pairwise comparisons.

Comparisons (group 1 versus group 2)	Numbers of DMRs*		Locations of DMRs relative to CpG islands (%)			
	**Group 1>group 2**	**Group 1<group 2**	**Islands**	**Shores**	**Shelves**	**Open seas**
Blast versus LSC	2,769	261	27.8	37.8	5.4	29
HSC versus MPP	14	2	33.3	0	0	66.7
MPP versus CMP	158	8	3.6	22.3	10.2	63.9
CMP versus GMP	366	362	2.5	17.0	15.4	65.1
CMP versus MEP	319	13	3.6	25.6	16.0	54.8
GMP versus MEP	1,308	764	2.4	19.3	15.3	63
MPP versus L-MPP	49	54	2.9	15.5	19.4	62.1
HSC versus L-MPP	165	109	2.2	19.0	15.0	63.7
L-MPP versus GMP	556	0	2.2	15.5	13.3	69.1
HSC versus GMP	1,168	162	2.0	17.8	13.5	66.7
HSC versus MEP	1,545	190	2.5	23.7	14.9	58.9

^*^P value cutoff of 0.01 (permutation test) was used to calculate the number of DMRs.

**Table 2:** Multivariate analysis of overall survival of TCGA patients using either DNA methylation or gene expression.

Variable	DNA methylation		Gene expression	
	**HR (95% CI)**	***P***	**HR (95% CI)**	***P***
Group	1.9 (1.2–2.9)	0.003	1.7 (1.0–2.7)	0.03
Age	1.04 (1.03–1.06)	8.5 × 10^−7^	1.04 (1.02–1.06)	1 × 10^−6^
				
*Cytogenetic risk*				
Intermediate versus low	2.7 (1.3–5.2)	0.005	2.2 (1.0–4.5)	0.04
High versus low	2.7 (1.3–5.6)	0.006	2.2 (1.0–4.7)	0.05
NPM1	0.8 (0.5–1.3)	0.39	1.0 (0.6–1.7)	1.00
FLT3	1.7 (1.1–2.8)	0.03	1.5 (0.9–2.5)	0.10

Log-rank test was used to assign statistical significance.

**Table 3:** Multivariate analysis of overall survival of patients in various cohorts using gene expression.

Variable	Metzeler *et al*.		Wouters *et al*.		Wilson *et al*.	
	**HR (95% CI)**	***P***	**HR (95% CI)**	***P***	**HR (95% CI)**	***P***
LSC score (high versus low)	1.6 (1.0–2.4)	4 × 10^−2^	1.8 (1.2–2.6)	3 × 10^−3^	1.8 (1.2–2.6)	5 × 10^−3^
Age	1.03 (1.01–1.04)	4 × 10^−4^	1.02 (1.0–1.03)	3 × 10^−2^	1.02 (1.01–1.04)	2 × 10^−3^
						
*Cytogenetics*						
Intermediate versus low	—	—	2.4 (1.4–4.2)	2 × 10^−3^	1.4 (0.6–3.3)	3.9 × 10^−1^
High versus low	—	—	3.1 (1.6–5.9)	7 × 10^−4^	3.0 (1.2–7.1)	2 × 10^−2^
FLT3	2.3 (1.5–3.6)	1 × 10^−4^	1.8 (1.3–2.6)	2 × 10^−3^	1.6 (1.0–2.5)	4 × 10^−2^
NPM1	0.7 (0.5–1.1)	8 × 10^−2^	0.5 (0.3–0.7)	2 × 10^−4^	0.7 (0.5–1.1)	1.2 × 10^−1^

Log-rank test was used to assign statistical significance.
