# Supplementary material for: An LSC epigenetic signature is largely mutation independent and implicates the HOXA cluster in AML pathogenesis
Source: Nat Commun. 2015 Oct 7;6:8489. doi: 10.1038/ncomms9489 (PMC4633733; doi:10.1038/ncomms9489)
Supplement: Supplementary Information — Supplementary Figures 1-8, Supplementary Tables 1-16 and Supplementary References [file ncomms9489-s1.pdf]

a

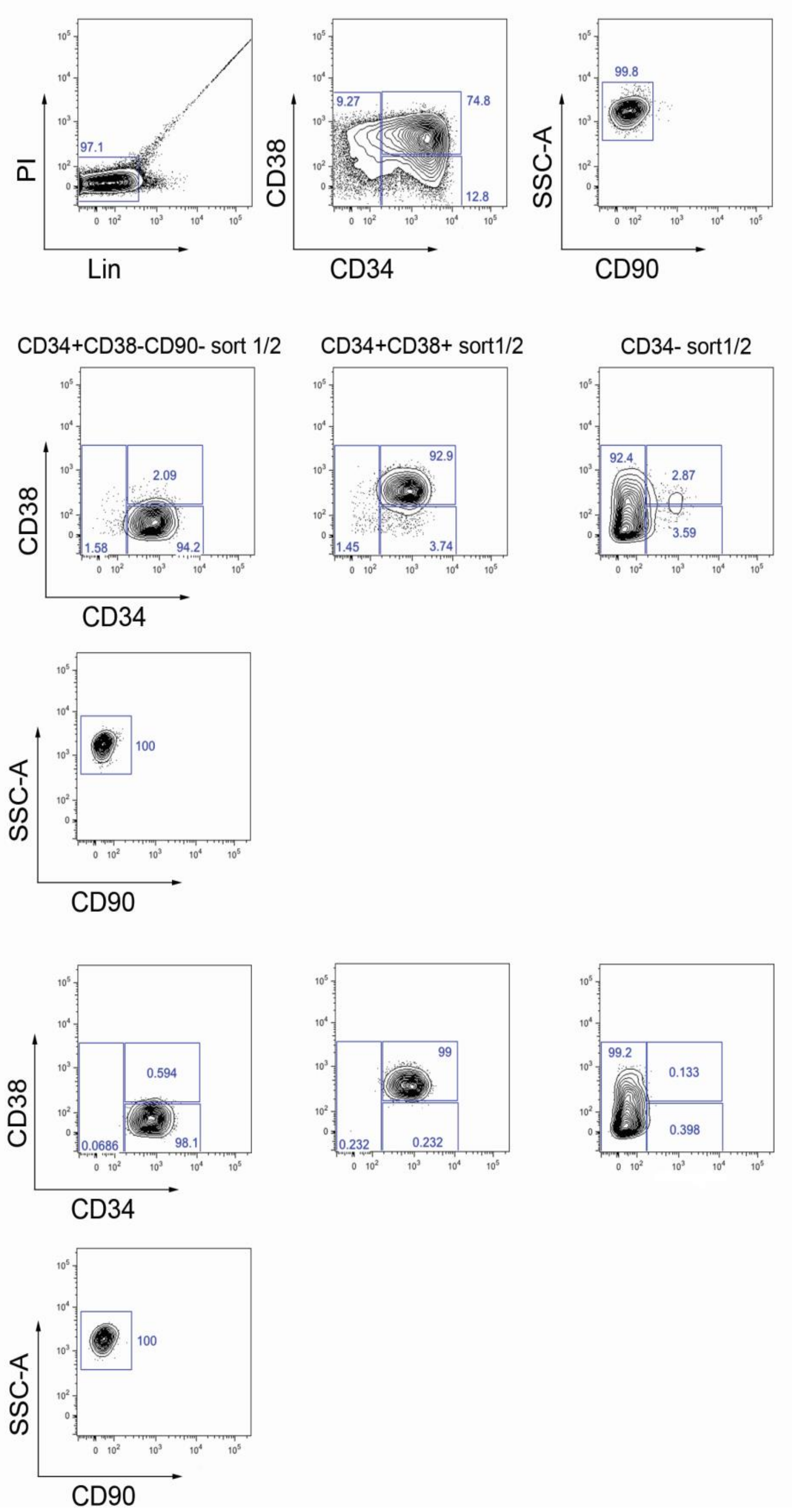
PI
Lin
97.1
CD38
CD34
9.27
74.8
12.8
SSC-A
CD90
99.8
CD34+CD38-CD90- sort 1/2
CD34+CD38+ sort1/2
CD34- sort1/2
2.09
1.58
94.2
92.9
1.45
3.74
92.4
2.87
3.59
100
0.594
0.0686
98.1
99
0.232
0.232
99.2
0.133
0.398
100

b

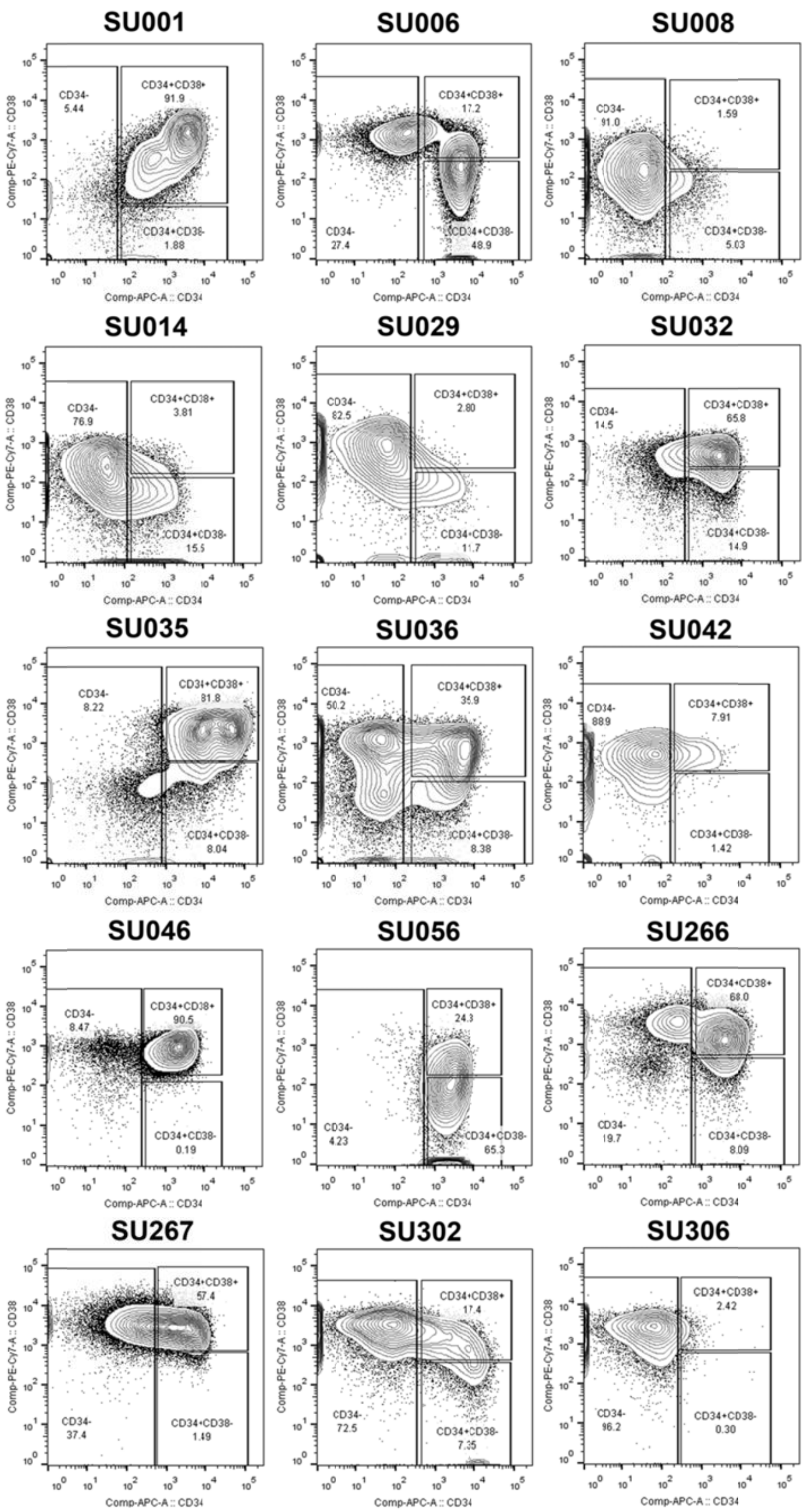

SU001
CD34- 5.44
CD34+CD38+ 91.9
CD34+CD38- 1.88
SU006
CD34+CD38+ 17.2
CD34- 27.4
CD34+CD38- 48.9
SU008
CD34- 91.0
CD34+CD38+ 1.59
CD34+CD38- 5.03
SU014
CD34- 76.9
CD34+CD38+ 3.81
CD34+CD38- 15.5
SU029
CD34- 82.5
CD34+CD38+ 2.80
CD34+CD38- 11.7
SU032
CD34- 14.5
CD34+CD38+ 65.8
CD34+CD38- 14.9
SU035
CD34- 8.22
CD34+CD38+ 81.8
CD34+CD38- 8.04
SU036
CD34- 50.2
CD34+CD38+ 35.9
CD34+CD38- 8.38
SU042
CD34- 88.9
CD34+CD38+ 7.91
CD34+CD38- 1.42
SU046
CD34- 8.47
CD34+CD38+ 90.5
CD34+CD38- 0.19
SU056
CD34+CD38+ 24.3
CD34- 4.23
CD34+CD38- 65.3
SU266
CD34+CD38+ 68.0
CD34- 19.7
CD34+CD38- 8.09
SU267
CD34+CD38+ 57.4
CD34- 37.4
CD34+CD38- 1.49
SU302
CD34+CD38+ 17.4
CD34- 72.5
CD34+CD38- 7.35
SU306
CD34+CD38+ 2.42
CD34- 96.2
CD34+CD38- 0.30
Comp-PE-Cy7-A :: CD38
Comp-APC-A :: CD34

Supplementary Figure 1. Pre-sort and post-sort FACS analysis of subpopulations from human AML. (**a**) Top panel: FACS-sorting scheme of three immunophenotypically defined subpopulations from human AML samples. Other panels: Two rounds of post-sort analysis to check the purity of sorting. (**b**) FACS sorting plot of all AML samples in this study.

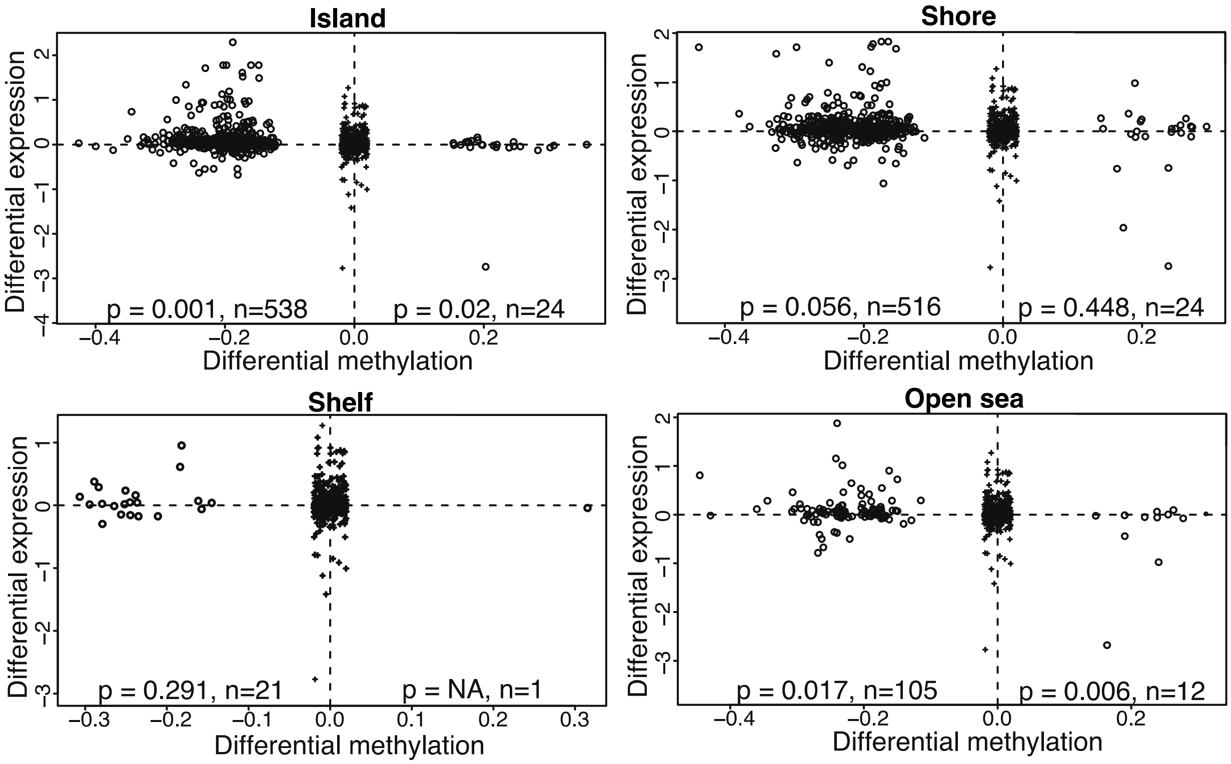

Supplementary Figure 2. Gene expression inversely correlates with DMRs at CpG island and open sea. Engrafting (LSC) and non-engrafting (blast) subpopulations from primary AML cases were profiled for DNA methylation and gene expression to identify differentially methylated regions (DMRs) and differentially expressed genes between these two groups. DMRs that are located within 2kb of gene transcriptional start sites (TSSs - black dots) were classified into 4 groups according to their distance relative to a CpG island: island, shore, shelf, and open sea. DMRs located further than 2kb away from TSSs are denoted as black pluses. Log_2 ratios of differential expression were plotted against differential methylation (all values are blast compared to LSC). Wilcoxon rank-sum tests were performed to test the null hypothesis that the expression differences for the hypo- or hypermethylated DMRs within 2kb of gene TSSs (black dots) showed stronger inverse correlation than the expression differences of the random DMRs that are located further than 2kb of TSSs (black pluses). Random DMRs were shown in the middles of DNA methylation axis regardless of their methylation differences.

a

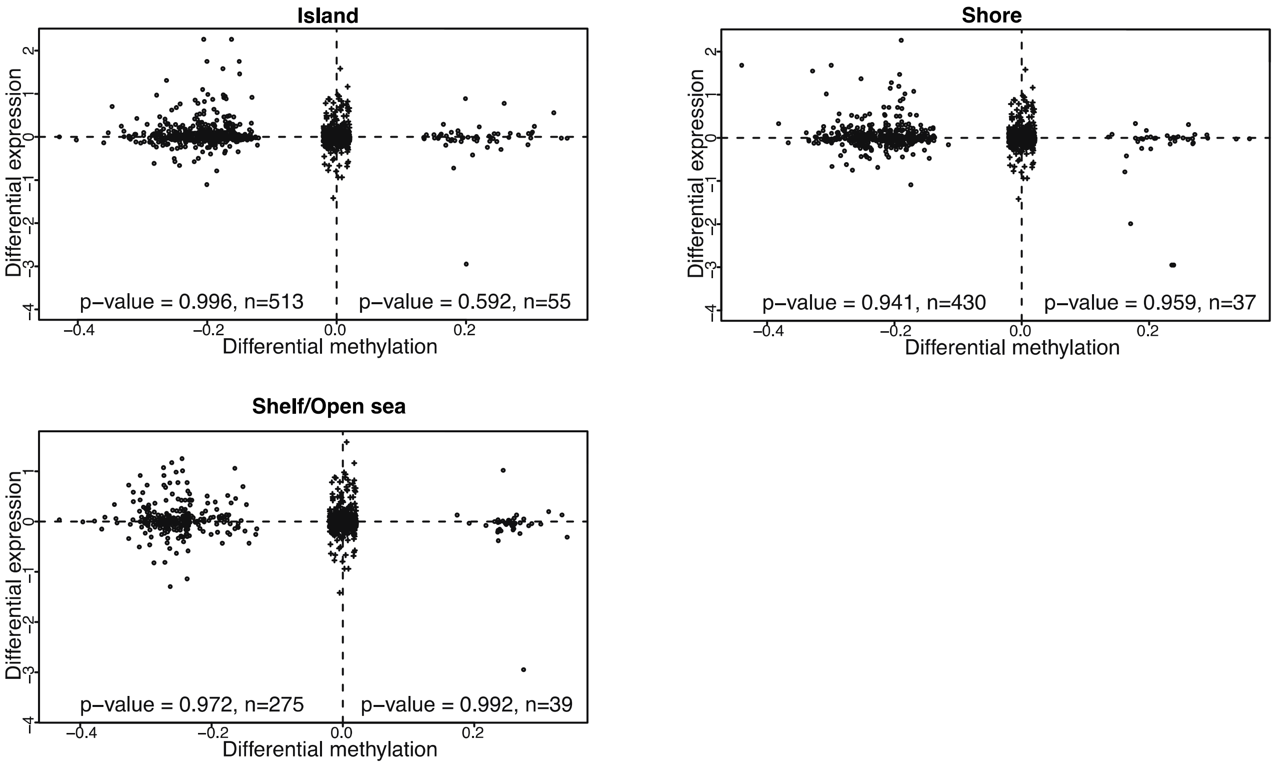
Island
Differential expression
Differential methylation
p−value = 0.996, n=513
p−value = 0.592, n=55
Shore
Differential expression
Differential methylation
p−value = 0.941, n=430
p−value = 0.959, n=37
Shelf/Open sea
Differential expression
Differential methylation
p−value = 0.972, n=275
p−value = 0.992, n=39

b

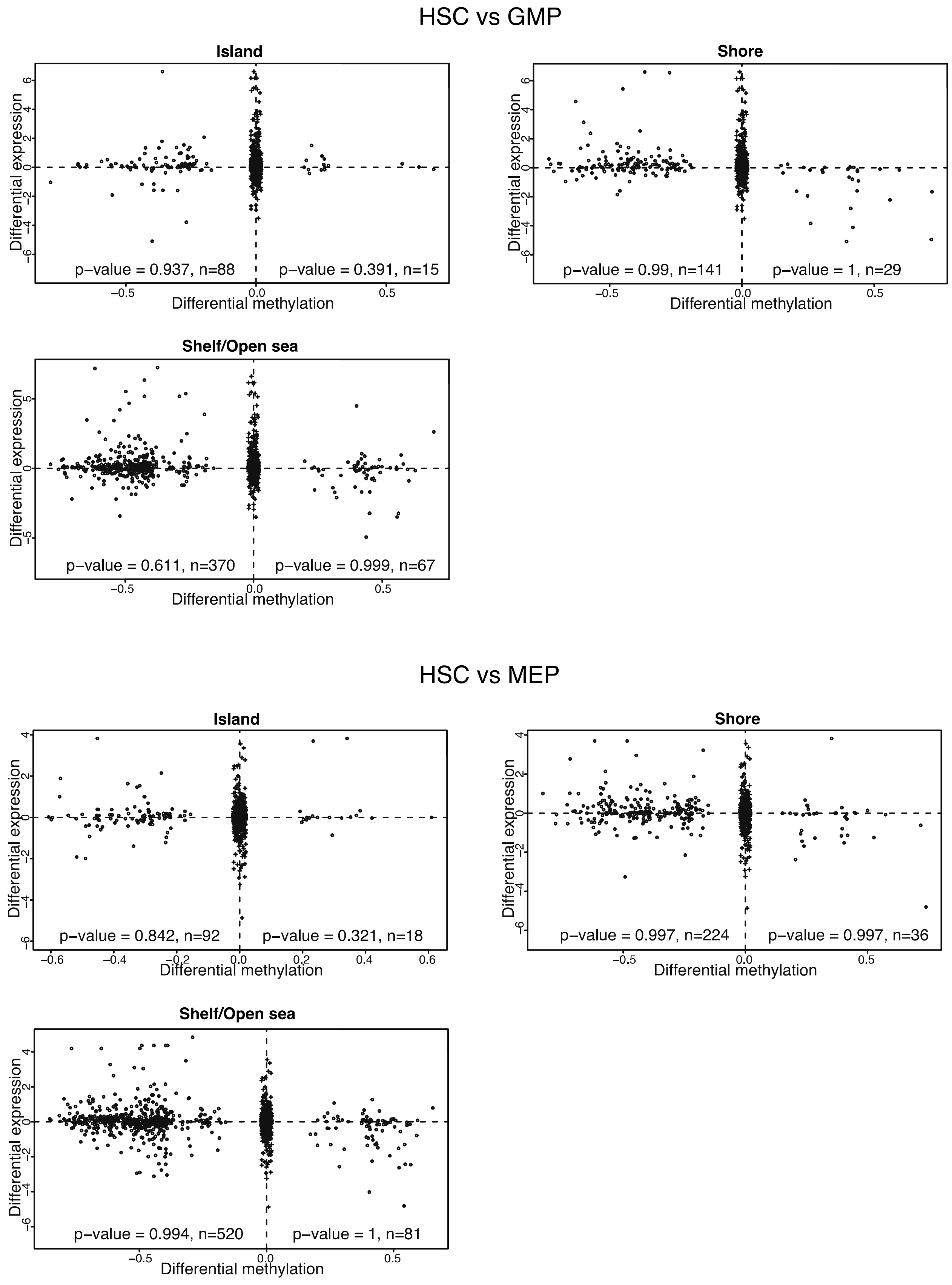
HSC vs GMP
Island
p−value = 0.937, n=88
p−value = 0.391, n=15
Shore
p−value = 0.99, n=141
p−value = 1, n=29
Shelf/Open sea
p−value = 0.611, n=370
p−value = 0.999, n=67
HSC vs MEP
Island
p−value = 0.842, n=92
p−value = 0.321, n=18
Shore
p−value = 0.997, n=224
p−value = 0.997, n=36
Shelf/Open sea
p−value = 0.994, n=520
p−value = 1, n=81
Differential expression
Differential methylation

Supplementary Figure 3. Gene body methylation doesn't show statistically significant positive correlation with gene expression. DMRs that are located in gene body (TSS to transcription end site (TES)) were classified into three groups according to their distance relative to a CpG island: island, shore, shelf/open sea. Random DMRs that don't locate in gene body are denoted as black pluses. Log_2 ratios of differential expression were plotted against differential methylation. Wilcoxon rank-sum tests were performed to test the null hypothesis that the expression differences for the hypo- or hypermethylated DMRs located in gene body (black dots) showed stronger positive correlation than the expression differences of the random DMRs that do not locate in gene body (black pluses). (**a**) LSC vs Blast. All values for DNA methylation and gene expression are from LSC-Blast. (**b**) Normal hematopoiesis. HSC vs GMP and HSC vs MEP are shown. All values for DNA methylation and gene expression are from group2 – group1 for group1 vs group2 comparisons.

a

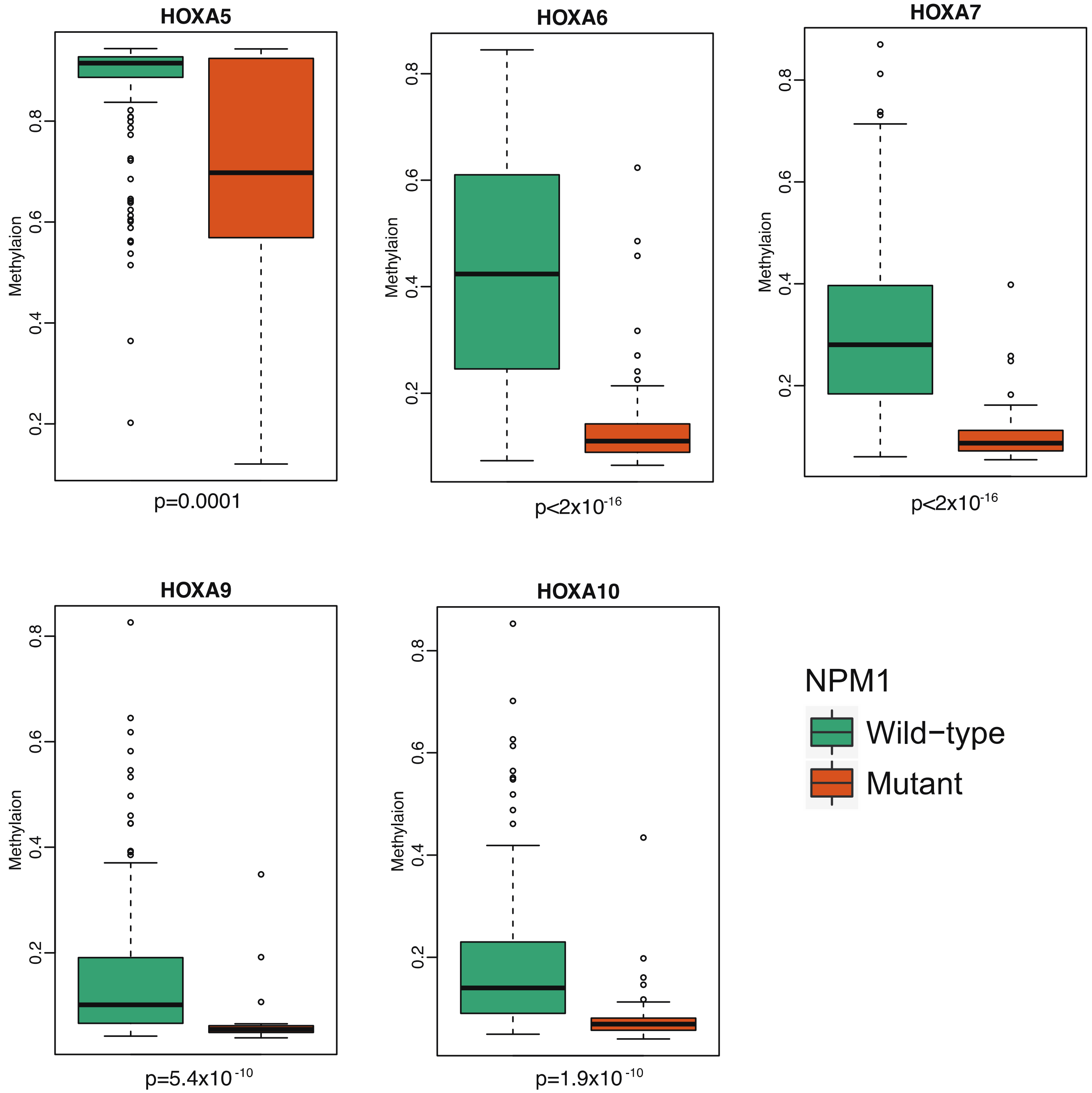
HOXA5
HOXA6
HOXA7
HOXA9
HOXA10
Methylaion
0.2
0.4
0.6
0.8
p=0.0001
p<2x10-16
p<2x10-16
p=5.4x10-10
p=1.9x10-10
NPM1
Wild−type
Mutant

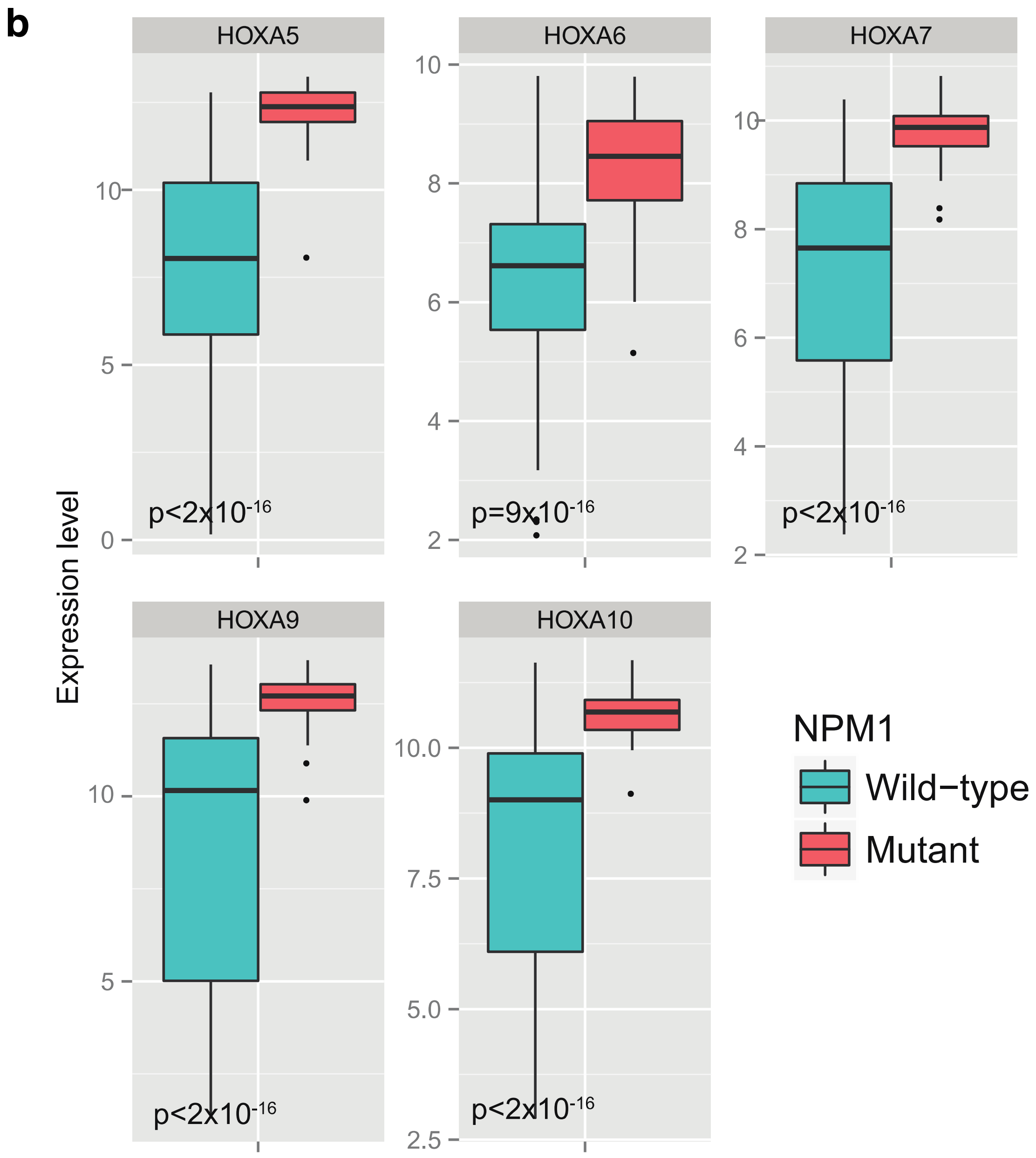

Supplementary Figure 4. *NPM1* mutation is associated with decreased methylation and increased expression of *HOXA* genes. Box plots are showing the rectangle of 1st quartile to 3rd quartile with median value as a horizontal line. The whiskers are ranged from the minimum to the maximum value (methylation or expression value) excluding the outliers. (**a**) Box plots show methylation level for *NPM1* mutants and wild-type samples for DMRs for *HOXA5*, *HOXA6*, *HOXA7*, *HOXA9,* and *HOXA10* in the TCGA dataset. t-test assuming unequal variance was performed to look at statistical significance of the association between *NPM1* mutation and methylation. DNA methylation of all the *HOXA* genes was significantly associated with *NPM1* mutation. (**b**) Box plots show gene expression (Log_2 value) for *NPM1* mutants and wild-type samples for *HOXA5*, *HOXA6*, *HOXA7*, *HOXA9,* and *HOXA10* in the TCGA dataset. t-test assuming unequal variance showed *NPM1* mutation highly correlated with increased expression of all the *HOXA* genes tested.

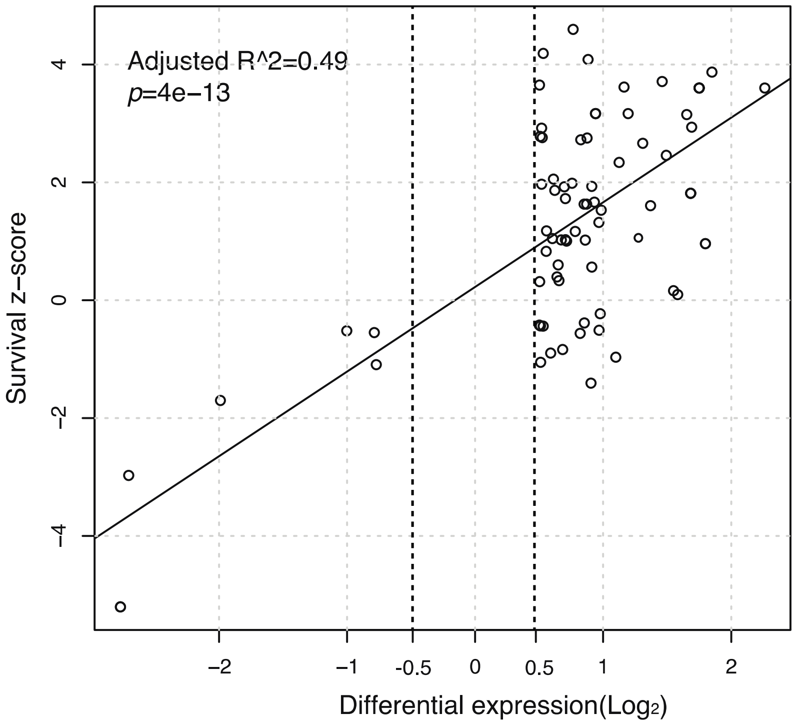

Supplementary Figure 5. The gene expression of the LSC epigenetic signature highly correlates with clinical outcome in the TCGA dataset. Each dot represents an LSC epigenetic signature gene. Survival z-score was plotted against log_2 ratio of differential expression of the LSC epigenetic signature genes in TCGA.

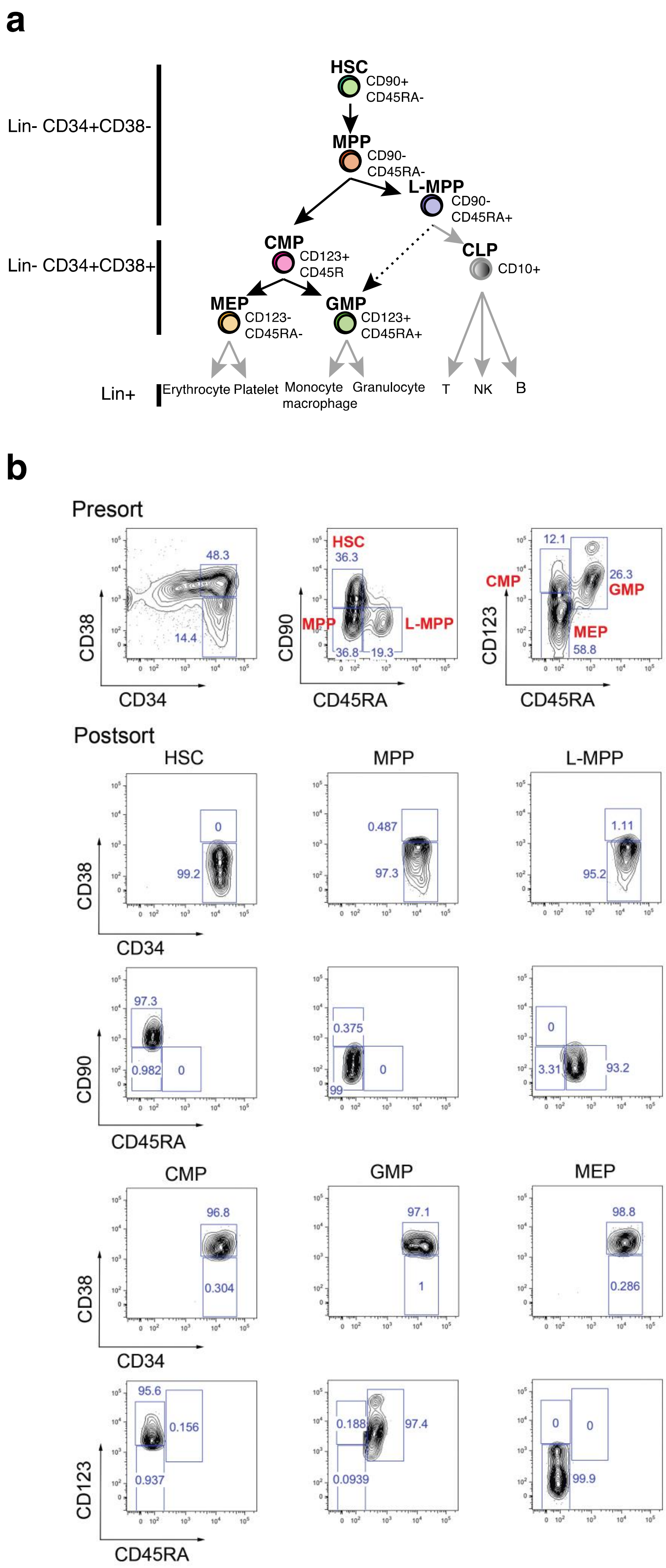
a
HSC
CD90+
CD45RA-
Lin- CD34+CD38-
MPP
CD90-
CD45RA-
L-MPP
CD90-
CD45RA+
CMP
CD123+
CD45R
CLP
CD10+
Lin- CD34+CD38+
MEP
CD123-
CD45RA-
GMP
CD123+
CD45RA+
Lin+
Erythrocyte Platelet
Monocyte
macrophage
Granulocyte
T
NK
B
b
Presort
CD38
CD34
48.3
14.4
CD90
CD45RA
HSC
36.3
MPP
L-MPP
36.8
19.3
CD123
CD45RA
12.1
CMP
26.3
GMP
MEP
58.8
Postsort
HSC
MPP
L-MPP
0
99.2
0.487
97.3
1.11
95.2
97.3
0.982
0
0.375
99
0
0
3.31
93.2
CMP
GMP
MEP
96.8
0.304
97.1
1
98.8
0.286
95.6
0.156
0.937
0.188
97.4
0.0939
0
0
99.9

Supplementary Figure 6. Pre-sort and post-sort FACS analysis of HSPCs from human bone marrows. (a) Schematic of human hematopoiesis with the immunophenotype of individual HSPC populations as indicated. Note the color scheme for each HSPC population is used throughout.(b) Pre-sort and post-sort FACS analysis of HSPCs from human bone marrow**.** Top panel: FACS-sorting scheme of six populations of HSPCs from normal human BM. Other panels: The second round of post-sort analysis to check the purity of sorting.

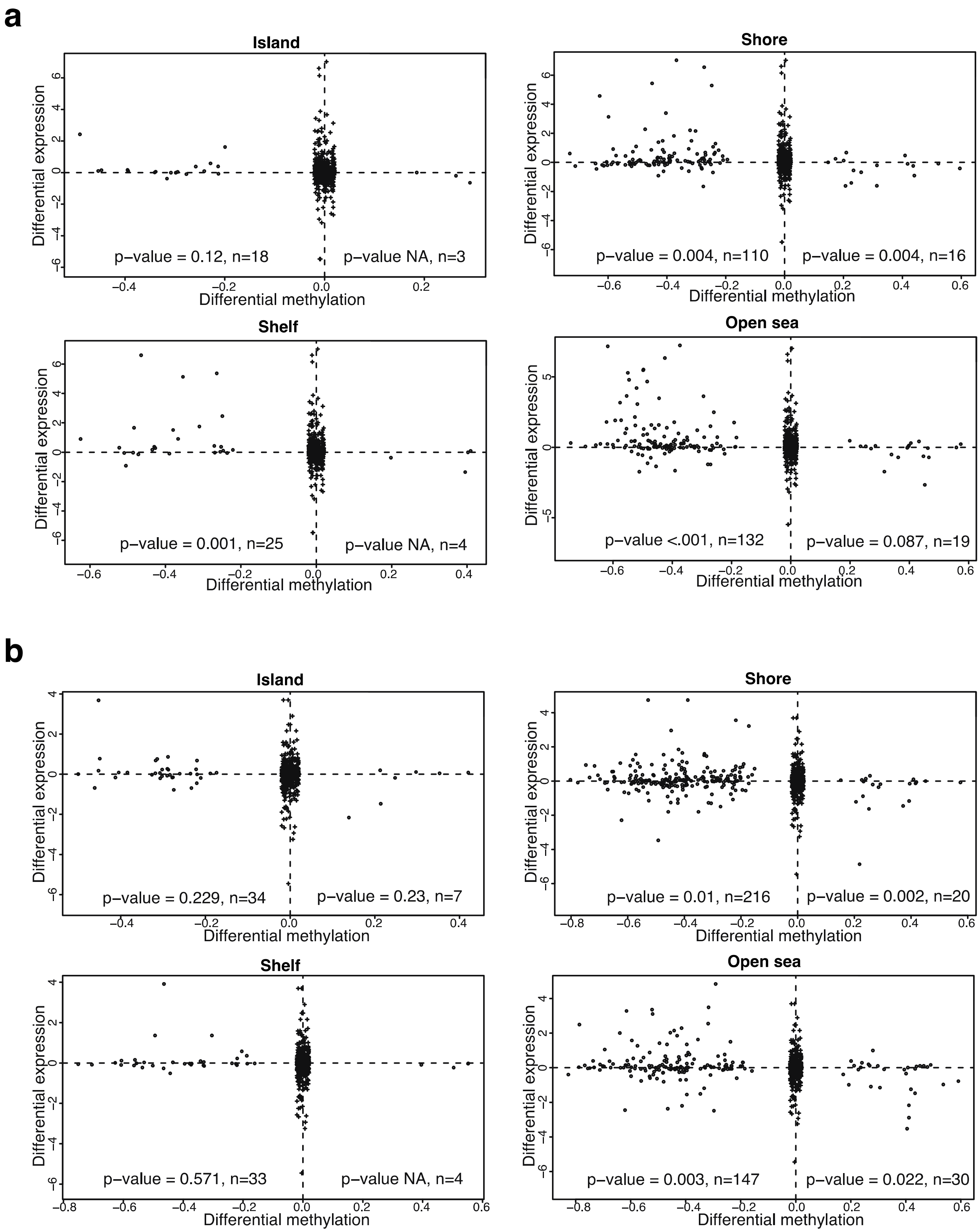
a
Island
Shore
Shelf
Open sea
Differential expression
Differential methylation
p−value = 0.12, n=18
p−value NA, n=3
p−value = 0.004, n=110
p−value = 0.004, n=16
p−value = 0.001, n=25
p−value NA, n=4
p−value <.001, n=132
p−value = 0.087, n=19
b
Island
Shore
Shelf
Open sea
p−value = 0.229, n=34
p−value = 0.23, n=7
p−value = 0.01, n=216
p−value = 0.002, n=20
p−value = 0.571, n=33
p−value NA, n=4
p−value = 0.003, n=147
p−value = 0.022, n=30

Supplementary Figure 7. Gene expression inversely correlates with DMRs at non-CpG island regions in normal hematopoiesis. DMRs located within 2kb of gene TSSs (black dots) were classified into 4 groups according to the distance relative to CpG island: island, shore, shelf, and open sea. DMRs located further than 2kb of gene TSSs are denoted as black pluses in the middle. Log2 ratios of differential expression were plotted against differential methylation (all values are from group2-group1). Wilcoxon rank-sum test was performed to test the null hypothesis that the expression differences for the hypo- or hypermethylated DMRs within 2kb of gene TSSs (black dots) showed stronger inverse correlation than the expression differences of the random DMRs that are located further than 2kb of TSSs (black pluses). Random DMRs were shown in the middles of DNA methylation axis regardless of their methylation differences. (**a**) For HSC vs GMP, shore showed statistically inverse correlation of DMR with gene expression. (**b**) For HSC vs MEP, shore and open sea showed statistically inverse correlation of DMR with gene expression.

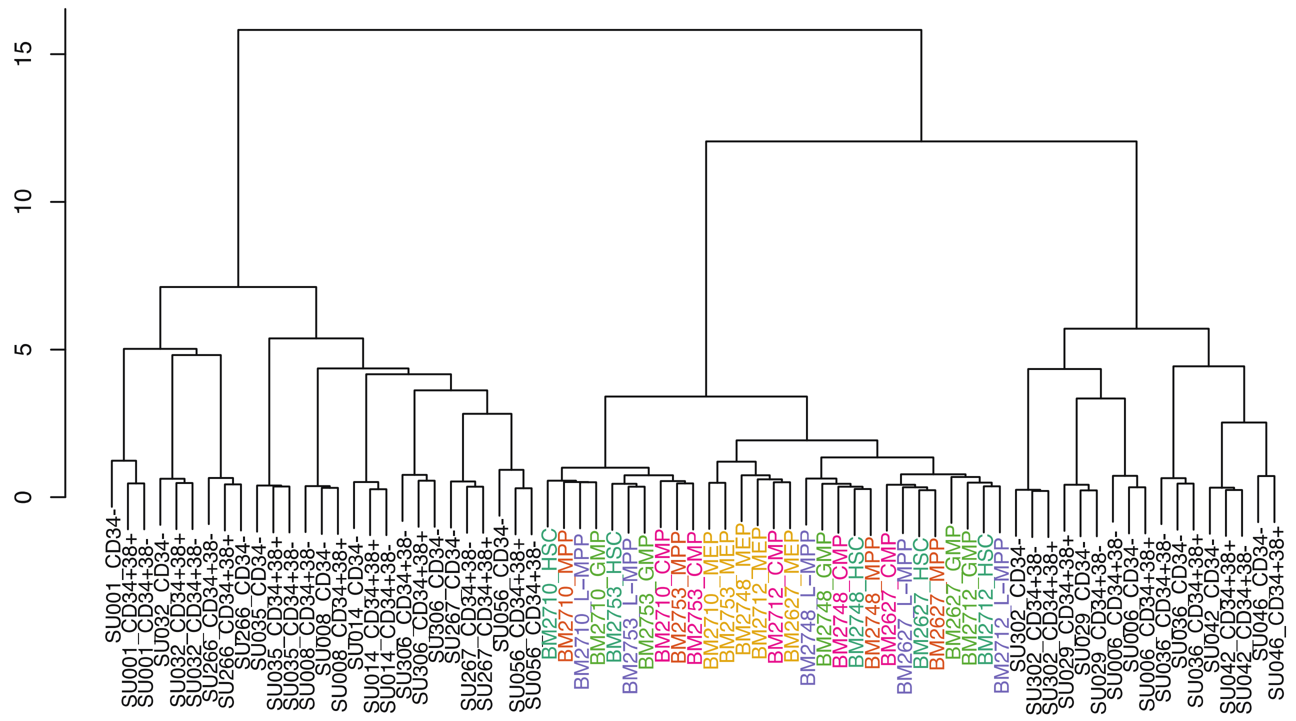

Supplementary Figure 8. Clustering analysis of AML populations with normal HSPCs using length matched random 216 regions. Clustering analysis using random length matched regions shows no clustering between AML populations or normal HSPCs. Normal progenitors clustered together, but not by lineages.

Supplementary Table 1. Clinical features of AML patients in study

Sample ID	Age	Gender	1° /2°	D/R	Cytogenetics	% CD34+	WHO Classification	FAB
SU001	59	F	1°	R	Normal	99	AML-not otherwise specified	M2
SU006	51	F	1°	D	Failed to grow	94	AML-not otherwise specified	M1
SU008	64	M	1°	D	Normal	3	AML-not otherwise specified	M1
SU014	59	M	1°	D	Normal	18	AML-not otherwise specified	ND
SU029	65	F	1°	D	inv(9)(p11q13)	8	AML with multilineage dysplasia without antecedent MDS	M2
SU032	47	M	1°	D	Normal	68	AML-not otherwise specified	M5
SU035	46	M	1°	D	Failed to grow	98	AML-not otherwise specified	M5
SU036	71	F	1°	D	t(8;21)	47	AML with t(8;21)(q22;q22)	ND
SU042	61	F	1°	D	t(10;11)	8	AML with 11q23 (MLL)	M5b
SU046	53	F	1°	D	t(6;11)	94	AML with 11q23 (MLL)	M5
SU056	56	M	1°	D	Complex cytogenetics	99	AML with multilineage dysplasia without antecedent MDS	M0
SU266	65	M	1°	D	inv(3)	96	AML with inv(3)(q21q26)	ND
SU267	58	M	1°	D	Normal	66	AML with multilineage dysplasia without antecedent MDS	ND
SU302	59	M	1°	D	Normal	14	AML-not otherwise specified	ND
SU306	33	F	1°	D	No analyzable metaphases	<1	AML-not otherwise specified	M5a

Abbreviations: 1° , primary; 2° , secondary; D, de novo; F, female; M, male; ND, no data; R, relapsed

Supplementary Table 2. Genetic mutations identified

Patient ID	TET2	IDH1	IDH2	DNMT3A	FLT3 ITD	FLT3 TKD	NPM1	KIT	CEBPA
SU001	wt	wt	wt	wt	wt	nd	wt	nd	nd
SU006	wt	wt	wt	wt	wt	nd	wt	nd	nd
SU008	wt	wt	wt	wt	mut	wt	wt	nd	nd
SU014	wt	R132H	wt	wt	mut	nd	mut	nd	nd
SU029	1149FS	wt	wt	R882H	mut	nd	mut	nd	nd
SU032	Y1649C	wt	wt	wt	wt	nd	wt	nd	nd
SU035	wt	wt	wt	wt	wt	nd	wt	nd	nd
SU036	wt	wt	wt	wt	nd	nd	wt	mut	nd
SU042	wt	wt	wt	S837*	wt	nd	wt	nd	nd
SU046	wt	wt	wt	wt	wt	wt	wt	nd	Nd
SU056	wt	wt	wt	wt	wt	wt	wt	nd	wt
SU266	E1010D	wt	wt	wt	wt	wt	wt	nd	wt
SU267	wt	R132C	wt	R882H	wt	wt	wt	nd	wt
SU302	wt	wt	wt	R882H	wt	wt	mut	wt	mut
SU306	wt	wt	R140Q	ΔV149	wt	mut	mut	wt	wt

Abbreviations: FS, frameshift mutation; wt, wild type; mut, mutant; nd, no data; * stop; Δ, deletion.

Note: Sanger sequencing was performed on TET2 exon 3-11, IDH1, IDH2 exon 4, and DNMT3A exon 3-11. More details are provided in Supplementary Table 21. For all other mutations, data are derived from clinical laboratory testing.

Supplementary Table 3. Engraftment of AML Subpopulations

Patient ID	"CD34-"	"CD34+CD38+"	"CD34+CD38-"
SU001	No	No	No
SU006	No	No	Yes
SU008	No	No	No
SU014	No	No	No
SU029	Yes	Yes	Yes
SU032	No	No	No
SU035	Yes	No	Yes
SU036	No	No	No
SU042	Yes	Yes	Yes
SU046	Yes	Yes	ND
SU056	No	Yes	Yes
SU266	No	Yes	Yes
SU267	No	Yes	Yes
SU302	No	Yes	Yes
SU306	No	No	Yes
Frequency	4/15 (26.7%)	7/15 (46.7%)	9/14 (64.3%)

Note: Yes: engrafted; No: no-engraftment; ND, no data. For SU046, there is no CD34+CD38- cell fraction.

Supplementary Table 4. Second DMR analysis to examine confounding effect of MLL cases

	LSC epigenetic signature (gene)	DMRs (p value<0.01)
**All Samples**	71	3030
**No MLL cases**	45	1398
**Overlap**	73.5%	77%

Supplementary Table 5. Univariate overall survival analysis for LSC epigenetic signature regarding differential gene expression in various cohorts

	TCGA		Metzeler et al		Wouters et al		Wilson et al	
Variable	HR (95% CI)	*p*	HR (95% CI)	*p*	HR (95% CI)	*p*	HR (95% CI)	*p*
LSC score (High vs. Low)	2.4 (1.6-3.6)	$1x10^{-5}$	1.9 (1.3-2.8)	$1 x 10^{-3}$	2.3 (1.7-3.1)	$2x10^{-7}$	2.2 (1.6-3.1)	$2x10^{-6}$
Age	1.04 (1.03-1.06)	$1x10^{-9}$	1.03 (1.01-1.04)	$3 x 10^{-4}$	1.01 (1.0-1.03)	$3 x 10^{-2}$	1.03 (1.02-1.05)	$4x10^{-6}$
Cytogenetics								
Intermediate vs. low	2.7 (1.4-5.2)	$2 x 10^{-3}$	-	-	2.8 (1.7-4.7)	$3x10^{-5}$	1.9 (0.9-4.1)	$1.1 x 10^{-1}$
High vs. low	3.9 (1.9-7.8)	$2 x 10^{-4}$	-	-	4.7 (2.7-8.4)	$1x10^{-7}$	4.2 (1.9-9.6)	$6x 10^{-4}$
FLT3	1.1 (0.7-1.7)	$7.1 x 10^{-1}$	2.2 (1.5-3.3)	$8x10^{-5}$	1.8 (1.3-2.5)	$5 x 10^{-4}$	1.1 (0.8-1.6)	$5.2 x 10^{-1}$
NPM1	1.4 (0.9-2.1)	$1.8 x 10^{-1}$	0.8 (0.5-1.2)	$2.4 x 10^{-1}$	0.9 (0.6-1.3)	$5.1 x 10^{-1}$	0.8 (0.5-1.1)	$2.0 x 10^{-1}$

Log-rank test was used to assign statistical significance

Supplementary Table 6. Univariate overall survival analysis for genetic mutations in epigenome modifying enzymes in TCGA

Genetic mutation	HR (95% CI)	*p*
DNMT3A	1.8 (1.2-2.7)	0.004
IDH1	0.8 (0.4-1.5)	0.4
IDH2	1.0 (0.6-1.9)	0.9
TET2	0.8 (0.4-1.7)	0.6
ASXL1	2.0 (0.6-6.4)	0.2

Log-rank test was used to assign statistical significance

Supplementary Table 7. Multivariate overall survival analysis including *DNMT3A* mutation for LSC epigenetic signature in TCGA

	DNA Methylation		Gene Expression	
Variable	HR(95% CI)	*p*	HR(95% CI)	*p*
Group	1.9 (1.2-3.0)	0.005	1.7 (1.0-2.7)	0.04
Age	1.0 (1.0-1.0)	9.3×10^{-7}	1.0 (1.0-1.0)	$1.2x10^{-6}$
Cytogenetic risk				
Intermediate/Normal	2.7 (1.3-5.6)	0.007	2.2 (1.0-4.6)	0.04
High	2.8 (1.3-5.9)	0.007	2.2 (1.0-4.8)	0.06
NPM1	0.8 (0.5-1.4)	0.46	1.0 (0.6-1.7)	0.99
FLT3	1.7 (1.0-2.8)	0.04	1.5 (0.9-2.5)	0.1
DNMT3	1.0 (0.6-1.6)	1.0	1.0 (0.7-1.6)	0.92

Log-rank test was used to assign statistical significance

Supplementary Table 8. Multivariate overall survival analysis for LSC epigenetic signature within intermediate cytogenetic risk patients in TCGA

	DNA Methylation		Gene Expression	
Variable	HR (95% CI)	*p*	HR (95% CI)	*p*
Group	1.8 (1.0-3.1)	0.05	1.9 (1.1-3.3)	0.03
Age	1.0 (1.0-1.1)	3×10^{-4}	1.0 (1.0-1.1)	2×10^{-4}
NPM1	0.7 (0.4-1.3)	0.2	0.9 (0.9-1.1)	0.7
FLT3	2.5 (1.3-4.8)	4×10^{-3}	2.3 (1.2-4.2)	0.01
DNMT3A	1.0 (0.6-1.8)	0.9	1.1 (0.7-1.9)	0.7

Log-rank test was used to assign statistical significance

Supplementary Table 9. Normal bone marrow donor sample analysis

Sample ID	Age	Gender	Application
BM2627	30	M	450K
BM2710	29	F	450K
BM2712	39	M	450K
BM2748	24	M	450K
BM2753	22	F	450K
BM2759	38	M	GEP
BM2761	26	M	GEP
BM2768	25	M	GEP
BM2770	39	F	GEP
BM2793	18	F	GEP
BM2794	21	M	GEP
BM2806	35	M	GEP
BM3604	26	M	P
BM3668	24	M	P
BM3671	24	M	P

Abbreviations: 450K, Illumina Infinium Human Methylation 450K BeadChip array; F, female; GEP, gene expression profiling microarray; M, male; P, bisulfite pyrosequencing

Supplementary Table 10. Antibodies for Flow Cytometry

Cell Surface marker	Fluorophore	Manufacturer	Catalog Number	Working Dilution	Application
CD2	PE-Cy5	BD Bioscience	555328	1:50	To sort normal HSPCs from BMs
CD3			555341		
CD4			555348		
CD7			555362		
CD8			555368		
CD10			555376		
CD11b			555389		
CD14			340585		
CD19			555414		
CD20			555624		
CD56			555517		
CD235a			559944		
CD34	APC		340667	1:50	
CD38	PE-Cy7		335790	1:100	
CD45RA	PB		560362	1:25	
CD90	FITC		555595	1:25	
CD123	PE		554529	1:25	
CD3	APC-Cy7	BD Bioscience	341090	1:50	To sort LSPCs from AML
CD19	PE-Cy5		555414		
CD20			555624		
CD34	APC		340667	1:50	
CD38	PE-Cy7		335790	1:100	
CD90	PE		555596	1:25	
CD3	APC-Cy7	BD Bioscience	341090	1:50	To test chimerism/ engraftment of LSC frequency in NSG mice
CD19	APC		555415	1:50	
CD33	PE		555450	1:50	
CD45	PB		560367	1:50	
CD45.1 (mouse)	PE-Cy7	eBioscience	25-0453-82	1:100	
Ter119 (mouse)	PE-Cy5		15-5921-83	1:100	

Supplementary Table 11. Multivariate overall survival analysis for FAB types

Variable	HR (95% CI)	*p*
<u>FAB types</u>		
M1 vs M0	1.5 (0.7-3.1)	0.31
M2 vs M0	2.1 (1.0-4.3)	0.06
M3 vs M0	1.0 (0.3-3.3)	0.99
M4 vs M0	1.3 (0.6-2.7)	0.49
M5 vs M0	2.1 (0.9-4.8)	0.07
M6 vs M0	2.5 (0.5-11.6)	0.24
M7 vs M0	2.1 (0.6-7.9)	0.25
Age	1.0 (1.0-1.1)	2.3×10^{-8}
<u>Cytogenetic risk</u>		
Intermediate vs low	2.8 (1.2-6.2)	0.01
High vs low	2.9 (1.2-6.8)	0.02
FLT3	1.8 (1.1-3.1)	0.02
NPM1	0.7 (0.4-1.2)	0.20
DNMT3A	1.1 (0.7-1.7)	0.72

Log-rank test was used to assign statistical significance

Supplementary Table 12. FAB type distribution for L-MPP-like and GMP-like AML samples

	M0	M1	M2	M3	M4	M5	M6	M7	NA
L-MPP	11	9	7	0	0	0	1	0	0
GMP	6	29	30	18	41	21	0	1	1

Supplementary Table 13. Cytogenetic risk group distribution for L-MPP-like and GMP-like samples

Cell identity	Favorable	Intermediate/Normal	Poor
GMP-like	33	90	22
L-MPP-like	0	13	15

Supplementary Table 14. Genetic mutation frequency for L-MPP-like and GMP-like AML samples

	L-MPP (%)	GMP (%)
DNMT3A	33.3	25.0
IDH1	33.3	6.3
IDH2	29.6	5.6
TET1	3.7	0.7
TET2	7.4	7.6
NPM1	3.7	34.7
FLT3	7.4	32.6

Supplementary Table 15. Primers for bisulfite pyrosequnecing

Gene	Primer type	Sequnece(5'->3')
MIR539	Nested forward	TATGATAAGTTTTGTAAAGGGATGTA
	Nested reverse	/5Biosg/CAAATCCCTAATAACACCAAAAAAT
	Long forward	GTGTTGTTGTTTTATATTTGAGGAGAA
	Long reverse	CATATCCAAAAAATACCTCCAAAAA
	Sequencing 1 (F)	TGATAAGTTTTGTAAAGGGATG
	Sequencing 2 (F)	GTTTTAATTTTAGAATTTTGGA
CDK6	Nested forward	TGTTTTTGAGATAGTAGTAGTAGGGTATTTTG
	Nested reverse	/5Biosg/TAACCAATCTAAACCCCATTTACTC
	Long forward	GGGGTAGATAGTTTTATATAGGGTAGTTGT
	Long reverse	TTCCACCCCAAAATTTATTATAACA
	Sequencing 1 (F)	GATAGTAGTAGGGTATTTTGAT
	Sequencing 2 (F)	ATTGTTTTTTTTTTGTTAAAGG
	Sequencing 3 (F)	TAAGTGGGAATTAAGTTTTGAG
HMHB1	Nested forward	TGGAGAAATTAGAATTGGAGGAGTA
	Nested reverse	/5Biosg/CTAAATAATCCCAACAACAAAAACC
	Long forward	ATGAGGAAATTATATTTTAGGAGGT
	Long reverse	CAACCAAACAATAAACTATAAAACC
	Sequencing 1 (F)	GAGAAGAAAAAAGAGGTGAGGG
	Sequencing 2 (F)	TATAATAGGTGAAAATAGGGAT
MPO	Nested forward	TAGTTTTAGTTGGTTGGATATGTTG
	Nested reverse	/5Biosg/AACCTCTCTCTATACCTCAAATCCC
	Long forward	TAGGTTGTTAAAGGGTAGTAGGGTT
	Long reverse	TACCAAAAATCCTAAAAACAAAAAA
	Sequencing 1 (F)	AGTTTTAGTTGGTTGGATATGT
	Sequencing 2 (F)	GTAGGTTTTTGGTTAGGGGTTT
	Sequencing 3 (F)	GGATGGTGATGTTGTT

/5Biosg/ = 5' biotin added
F=forward

Supplementary Table 16. Primers used for sequencing of *TET2*, *IDH1*, *IDH2* and *DNMT3A* mutations of AML

Primers	Sequence 5' to 3'	Size	Tm	Reference
(1) TET2 exon 3 PCR1 F	TGAACTTCCCACATTAGCTGGT	955	55	[1]
(2) TET2 exon 3 PCR1 R	GAAACTGTAGCACCATTAGGCATT			
(3) TET2 exon 3 PCR1 Seq	GATAGAAATAAACACATTTT			
(4) TET2 exon 3 PCR2 F	CAAAAGGCTAATGGAGAAAGACGTA	836	55	
(5) TET2 exon 3 PCR2 R	GCAGAAAAGGAATCCTTAGTGAACA			
(6) TET2 exon 3 PCR3 F	GCCAGTAAACTAGCTGCAATGCTAA	846	55	
(7) TET2 exon 3 PCR3 R	TGCCTCATTACGTTTTAGATGGG			
(8) TET2 exon 3 PCR4 F	GACCAATGTCAGAACACCTCAA	867	60	
(9) TET2 exon 3 PCR4 R	TTGATTTTGAATACTGATTTTCACCA			
(10) TET2 exon 3 PCR5 F	TTGCAACATAAGCCTCATAAACAG	788	60	
(11) TET2 exon 3 PCR5 R	ATTGGCCTGTGCATCTGACTAT			
(12) TET2 exon 3 PCR6 F	GCAACTTGCTCAGCAAAGGTACT	781	60	
(13) TET2 exon 3 PCR6 R	TGCTGCCAGACTCAAGATTTAAAA			
(14) TET2 exon 4 F	ATACTACATATAATACATTCTAATTCCCTCACTG	495	55	
(15) TET2 exon 4 R	TGTTTACTGCTTTGTGTGTGAAGG			
(16) TET2 exon 5 F	CATTTCTCAGGATGTGGTCATAGAAT	286	55	
(17) TET2 exon 5 R	CCCAATTCTCAGGGTCAGATTTA			
(18) TET2 exon 6 F	AGACTTATGTATCTTTCATCTAGCTCTGG	599	60	
(19) TET2 exon 6 R	ACTCTCTTCCTTTCAACCAAAGATT			
(20) TET2 exon 7 F	ATGCCACAGCTTAATACAGAGTTAGAT	362	55	
(21) TET2 exon 7 R	TGTCATATTGTTCACTTCATCTAAGCTAAT			
(22) TET2 exon 8 F	GATGCTTTATTTAGTAATAAAGGCACCA	354	55	
(23) TET2 exon 8 R	TTCAACAATTAAGAGGAAAAGTTAGAATAATATTT			
(24) TET2 exon 9 F	TGTCATTCCATTTTGTTTCTGGATA	361	55	
(25) TET2 exon 9 R	AAATTACCCAGTCTTGCATATGTCTT			
(26) TET2 exon 10 F	CTGGATCAACTAGGCCACCAAC	774	55	
(27) TET2 exon 10 R	CCAAAATTAACAATGTTCATTTTACAATAAGAG			
(28) TET2 exon 11 PCR1 F	GCTCTTATCTTTGCTTAATGGGTGT	748	60	
(29) TET2 exon 11 PCR1 R	TGTACATTTGGTCTAATGGTACAACTG			
(30) TET2 exon 11 PCR2 F	AATGGAAACCTATCAGTGGACAAC	1107	60	
(31) TET2 exon 11 PCR2 R	TATATATCTGTTGTAAGGCCCTGTGA			

(32) IDH1 exon 4 F	TGTGTTGAGATGGACGCCTATTTG	481	55	[2]
(33) IDH1 exon 4 R	TGCCACCAACGACCAAGTCA			
(34) IDH2 exon 4 F	GGGGTTCAAATTCTGGTTGA	290	53	
(35) IDH2 exon 4 R	CTAGGCGAGGAGCTCCAGT			
(36) DNMT3A exons 7-8 F	ATGGTCCCCTTGAGTGTCAG	836	56	[3]
(37) DNMT3A exons 7-8 R	CATCACCCCAATTCCAGACT			
(38) DNMT3A exons 9-10 F	CTGTATCTGGTCCCCTCCAG	747	56	
(39) DNMT3A exons 9-10 R	CTCCCTAAGCATGGCTTTCC			
(40) DNMT3A exons 11-12 F	GGGAACAAGTTGGAGACCAG	490	56	
(41) DNMT3A exons 11-12 R	GGTCCCATGTCATTCAAACC			
(42) DNMT3A exon 13 F	GTCACAGTGCCTCCCTTTTC	308	56	
(43) DNMT3A exon 13 R	TGGACACAGTCAGCCAGAAG			
(44) DNMT3A exon 14 F	CAGGGCTTAGGCTCTGTGAG	359	56	
(45) DNMT3A exon 14 R	AGGTGTGCTACCTGGAATGG			
(46) DNMT3A exons 15-16 F	CGGTCTTTCCATTCCAGGTA	614	56	
(47) DNMT3A exons 15-16 R	CATCATTTCGTTTTGCCAGA			
(48) DNMT3A exon 17 F	GACTTGGGCCTACAGCTGAC	345	58	
(49) DNMT3A exon 17 R	CAAAATGAAAGGAGGCAAGG			
(50) DNMT3A exons 18-19 F	CTTCCTGTCTGCCTCTGTCC	552	56	
(51) DNMT3A exons 18-19 R	ATGAAGCAGCAGTCCAAGGT			
(52) DNMT3A exons 19b-20 F	GCAGCACTGTGCAATATGGT	549	56	
(53) DNMT3A exons 19b-20 R	CTTCCCCACTATGGGTCATC			
(54) DNMT3A exons 21 F	GCGGGGAGTTTGAAGAGAGT	342	56	
(55) DNMT3A exons 21 R	CCACACTAGCTGGAGAAGCA			
(56) DNMT3A exons 22 F	TTTGGTAGACGCATGACCAG	301	56	
(57) DNMT3A exons 22 R	CAGGACGTTTGTGGAAAACA			
(58) DNMT3A exons 23 F	TCCTGCTGTGTGGTTAGACG	654	56	
(59) DNMT3A exons 23 R	CCTCTCTCCCACCTTTCCTC			
(60) DNMT3A exon 17 F	CCTCGATGTCCTTACTATGGATACTCCA	402	63	Additional primers were designed to cover the ones not working in previous rows. All the three new pairs worked on DNMT3A exon 17
(61) DNMT3A exon 17 R	CAAGGGCTGCCTCCAGGTGCTGAG		69	
(62) DNMT3A exon 17 F	CTCACCTGCCGAGACCAG	276	59	
(63) DNMT3A exon 17 R	CCTCCAGGTGCTGAGTGTG		60	
(48) DNMT3A exon 17 F	GACTTGGGCCTACAGCTGAC	437	60	
(64) DNMT3A exon 17 R	TTTGCCCTTTACCCTCTCAA		57	

Note: For IDH1 and IDH2, a single point mutation was tested in exon 4 (R132 and R140 respectively); for TET2 and DNMT3A mutations, multiple exons were tested based on regions of frequent somatic mutation according to COSMIC database (Wellcome Trust Sanger Institute).

SUPPLEMENTARY REFERENCES

1. Gelsi-Boyer, V., et al., *Mutations of polycomb-associated gene ASXL1 in myelodysplastic syndromes and chronic myelomonocytic leukaemia.* Br J Haematol, 2009. **145**(6): p. 788-800.
2. Thol, F., et al., *IDH1 mutations in patients with myelodysplastic syndromes are associated with an unfavorable prognosis.* Haematologica, 2010. **95**(10): p. 1668-74.
3. Fernandez-Mercado, M., et al., *Mutation patterns of 16 genes in primary and secondary acute myeloid leukemia (AML) with normal cytogenetics.* PLoS One, 2012. **7**(8): p. e42334.
