# Supplementary material for: An LSC epigenetic signature is largely mutation independent and implicates the HOXA cluster in AML pathogenesis
Source: Nat Commun. 2015 Oct 7;6:8489. doi: 10.1038/ncomms9489 (PMC4633733; doi:10.1038/ncomms9489)
Supplement: Supplementary Software 1 — R script for multivariate survival analysis [file ncomms9489-s8.docx]

a

## DNA methylation analysis and survival

stime <- ifelse(tpd$vital_status=="DECEASED",tpd$days_to_death,tpd$days_to_last_followup)

stime[stime%in%c("[Not Available]","[Not Applicable]")]<-NA

stime<-as.numeric(stime)

event<-tpd$vital_status=="DECEASED"

age<-tpd$age_at_initial_pathologic_diagnosis

prog<-tpd$acute_myeloid_leukemia_calgb_cytogenetics_risk_category

prog[prog=="[Not Available]"]<-NA

prog<-factor(prog,levels=c("Favorable","Intermediate/Normal","Poor"),labels=c("F","I","P"))

library(survival)

summary(coxph(Surv(stime,event)~group+age+prog+Flt3+Npm1)

summary(coxph(Surv(stime,event)~group+age+prog+Flt3+Npm1+tpd$dnmt3a)

b

## GEP analysis and survival

dmrexp = read.delim("TCGA_AML_newDMR_p001_fc05.eigengenes.pcl", stringsAsFactors=FALSE)

amlinfo2 = merge(amlinfo,dmrexp, by="Array")

medexp = median(amlinfo2$DMR_p0.01_fc0.5, na.rm=TRUE)

amlinfo2$medexp = 1

amlinfo2$medexp[amlinfo2$DMR_p0.01_fc0.5>medexp] = 2

summary(coxph(Surv(OS_Time,OS_Status) ~

DMR_p0.01_fc0.5+NPM+FLT3+dnmt3a+Age+CALGB_cytorisk, data=amlinfo2))

summary(coxph(Surv(OS_Time,OS_Status) ~

DMR_p0.01_fc0.5+NPM+FLT3+Age+CALGB_cytorisk, data=amlinfo2))

Supplementary Software 1. R script for multivariate survival analysis. (a)

Multivariate survival analysis for DNA methylation data in TCGA. The line or a variable

that show how we treated cytogenetic groups is colored in red. (b) Multivariate survival

analysis for gene expression data in TCGA. The line or a variable that show how we

treated cytogenetic groups is colored in red.
